# Inflammatory Joint Disease Is a Risk Factor for Streptococcal Sepsis and Septic Arthritis in Mice

**DOI:** 10.3389/fimmu.2020.579475

**Published:** 2020-10-07

**Authors:** Johann Volzke, Daniel Schultz, Marcel Kordt, Michael Müller, Wendy Bergmann, Karen Methling, Bernd Kreikemeyer, Brigitte Müller-Hilke, KoInfekt Study Group

**Affiliations:** ^1^Core Facility for Cell Sorting and Cell Analysis, University Medical Center Rostock, Rostock, Germany; ^2^Institute of Biochemistry, University of Greifswald, Greifswald, Germany; ^3^Institute of Medical Microbiology, Virology and Hygiene, University Medical Center Rostock, Rostock, Germany

**Keywords:** immunology, infection, sepsis, septic arthritis, group A streptococcus, rheumatoid arthritis, collagen-induced arthritis, bone erosion

## Abstract

Septic arthritis is a medical emergency associated with high morbidity and mortality, yet hardly any novel advances exist for its clinical management. Despite septic arthritis being a global health burden, experimental data uncovering its etiopathogenesis remain scarce. In particular, any interplay between septic arthritis and preceding joint diseases are unknown as is the contribution of the synovial membrane to the onset of inflammation. Using C57BL/6 mice as a model to study sepsis, we discovered that Group A Streptococcus (GAS) – an important pathogen causing septic arthritis - was able to invade the articular microenvironment. Bacterial invasion resulted in the infiltration of immune cells and detrimental inflammation. *In vitro* infected fibroblast-like synoviocytes induced the expression of chemokines (*Ccl2*, *Cxcl2*), inflammatory cytokines (*Tnf*, *Il6*), and integrin ligands (ICAM-1, VCAM-1). Apart from orchestrating immune cell attraction and retention, synoviocytes also upregulated mediators impacting on bone remodeling (*Rankl*) and cartilage integrity (*Mmp13*). Using collagen-induced arthritis in DBA/1 × B10.Q F1 mice, we could show that an inflammatory joint disease exacerbated subsequent septic arthritis which was associated with an excessive release of cytokines and eicosanoids. Importantly, the severity of joint inflammation controlled the extent of bone erosions during septic arthritis. In order to ameliorate septic arthritis, our results suggest that targeting synoviocytes might be a promising approach when treating patients with inflammatory joint disease for sepsis.

## Introduction

Streptococcus pyogenes (Group A Streptococcus, GAS) is a gram-positive pathogen causing a wide variety of usually non-severe disorders like impetigo and pharyngitis (1). However, GAS can also turn into a severe threat. Among many others, it uses lipoteichoic acid and the M protein as virulence factors to recognize host matrix molecules and thereby adheres to and breaches host tissue barriers (2–4). Moreover, GAS can proficiently subvert the immune response of the host by, for example, inducing apoptosis in immune cells (5) and degrading neutrophil extracellular traps (6). Thus, it causes severe bacteremia resulting in a broad collection of critical conditions (7). Indeed, invasive GAS infections are estimated to cause more than 18 million cases worldwide leading to 517,000 deaths and an approximated 1.78 million new cases per year (8). As there is no vaccine against this pathogen (9), therapies need to be improved which in turn requires a better understanding of the pathological manifestations leading to severe disease courses like streptococcal toxic shock syndrome, necrotizing fasciitis, and septic arthritis (SA).

GAS is the second leading cause of SA and responsible for roughly 8%–16% of all cases reported (10–13). SA includes GAS invading the joint and thereby stimulating a local immune response that in turn results in irreversible and aggressively progressing erosions of bone and cartilage. In fact, it has been estimated that 30 percent of all patients surviving SA suffer from chronic joint damage eventually causing invalidity (14, 15). The mortality of this disorder remains continuously high and research data defining the pathogenesis of SA are limited (11, 16–18). In particular, the significance of pre-existing joint disorders for the onset and progression of SA remains obscure, despite epidemiological studies pointing toward autoimmune joint diseases as risk factors (13, 16, 19, 20).

Indeed, treatment regimen for autoimmune diseases like rheumatoid arthritis (RA) often include immunosuppression and are therefore being considered an elevated risk for invasive bacterial infections (18). Likewise, the application of monoclonal anti-TNFα antibodies has been reportedly associated with aggravated infections (21, 22). However, alternative hypotheses imply that autoimmune disorders by themselves predispose for SA due to an impaired ability to resist infections (16, 23, 24).

In order to investigate both, autoimmunity as a facilitator of aggravated sepsis and pre-existing inflammatory joint disease as risk factors for septic arthritis, we here turned to the mouse model of collagen induced arthritis (CIA). CIA shares important homologies with rheumatoid arthritis like synovial hyperplasia and articular immune cell infiltration (25–27). Triggering CIA prior to GAS infection therefore allowed for the analysis of the immune response, bone metabolism and the contribution of the synovial lining of the joint and the stromal cells to the disease process of septic arthritis.

## Materials and Methods

### Animals

C57Bl/6J (B6) mice were initially purchased from Charles River (Wilmington, MA, USA). DBA/1 and B10.Q from Harlan Winkelmann (Borchen, Germany). The F1 generation resulting from a cross between female DBA/1 and male B10.Q mice was used for experiments. All strains were bred continuously in our animal care facility under specific germ-free conditions and were housed in individually ventilated cages at a 12-h light/dark cycle and an ambient temperature of 21 ± 2°C with 60 ± 10% humidity. Mice were given water and ssniff R/M-H diet (ssniff Spezialdiäten GmbH, Soest, Germany) ad libitum. Animal experiments were reviewed and approved by the federal state’s animal ethics committee (State Department for Agriculture, Food Safety and Fishery in Mecklenburg-Western Pomerania) with the file reference number 7221.3-1.1-063/17.

### Induction of Invasive Streptococcal Infection and Septic Arthritis

For all infection experiments, the *emm1* Group A Streptococcus (GAS) strain AP1 was used which was originally received from the World Health Organization Collaborating Center for Reference and Research on Streptococci (Prague, Czech Republic). Due to selection in the original human host, AP1 contains inactivating mutations in the *covS* gene that decrease repression of streptococcal virulence factors (28). As a result, AP1 is associated with invasive infections and is reportedly highly pathogenic in mice (29, 30). Bacteria were suspended into Todd-Hewitt broth (THB, Becton Dickinson, Franklin Lakes, NJ, USA) and cultured for about 16 h until stationary phase of growth was reached. A 20-fold dilution of the suspension was performed with THB and bacteria were cultured until mid-log phase of growth was attained as confirmed by an optical density (600 nm) of 0.4-0.6. Bacteria were washed twice and the OD was adjusted to 0.5 which corresponded to 0.9–1.1 × 10^8^ CFU/ml as confirmed by blood agar cultures. Mice were restrained and the tail was disinfected. 100 µl of the diluted bacterial suspension was then injected intravenously with a 30 G needle at doses of 0.5 × 10^6^ and 2.0 × 10^6^ bacteria for B6 and F1 mice, respectively. Mice were subsequently monitored for 14 days post infection (p.i.). Animals with severely progressed disease were sacrificed at strictly defined clinical endpoints (see *Clinical Scoring*). At these endpoints, mice were anaesthetized and blood was collected by cardiac puncture. Animals were subsequently sacrificed by cervical dislocation. Afterwards, a medial arthrotomy was performed on both knee joints and swabs of the synovial fluid were performed for bacterial load determination. Hind- and forelimbs were extracted for fixation in 4% formaldehyde (Formafix, Düsseldorf, Germany) and snap frozen for storage at -80°C, respectively. The spleen, half of the liver and whole blood were determined for bacterial load. The residual liver was snap frozen and stored at -80°C for further analysis. The remaining anticoagulated blood samples were used for separation of plasma.

### Collagen-Induced Arthritis

Male F1 mice at the age of 6–8 weeks were subjected to collagen-induced arthritis (CIA) by subcutaneous injection of 140 µg bovine type II collagen (Chondrex, Redmond, WA, USA) dissolved in 0.1 M acetic acid and emulsified with an equivalent volume of complete Freund’s adjuvant (Becton Dickinson). The injection site for the primary immunization was located 5 mm distal from the base of the tail. For secondary immunization 3 weeks later, 140 µg collagen was emulsified in incomplete Freund’s adjuvant and was applied slightly distal from the prior injection site. Mice were monitored for 11 weeks after the booster injection at which time point they were infected with GAS. For analgesia, mice were given Tramadol (Ratiopharm, Ulm, Germany) at a final concentration of 1 mg/ml with the drinking water.

### Clinical Scoring

Macroscopic scoring of hind and fore limbs for signs of arthritis was carried out every other day starting at boost. Severity of CIA was quantified as described previously (31): 1 point for redness and/or swelling for each affected digit, 5 points for redness and/or swelling for each affected palmar or plantar paw area and likewise, 5 points for redness and/or swelling for each affected wrist or ankle joint. Scoring for signs of septic arthritis was conducted once every day after GAS injection in a similar fashion. Additional 5 scoring points were added for each knee if bacteria were isolated from the joint capsule. The occurrence of an arthritis score of ≥ 1 was considered a disease incidence event for CIA and SA, alike. Severity of sepsis was scored at least once a day after bacterial infection using macroscopic burden manifestations as a proxy based on a previously described method (32) with few alterations. In brief, sepsis scoring was subdivided into four categories (weight loss, appearance, consciousness, respiration) each with a maximum score of 25 (**Table 1**). A sepsis score ≥ 5 was defined as sepsis occurrence. Animals with a total score of ≥ 25 were considered at septic shock (humane endpoint) and were sacrificed. Sacrifice at septic shock was implemented into the analysis of survival probability.

**Table 1:** Murine Sepsis Score for disease severity using signs of burden after Group A Streptococcus (GAS) infection as a proxy.

Category	Score and description
Relative body weight	0 - no reduction or increase 5 - reduction ≥ 5% 10 - reduction ≥ 10% 25 - reduction ≥ 20%
Appearance	0 - smooth and gleaming fur, clean body orifices 5 - piloerection, badly groomed body orifices, eyes cloudy 10 - clotty and moist orifices, high myotonos, eyes with secretions 25 - convulsions, paralysis, animal appears cold/moribund
Level of consciousness	0 - mouse is active, normal behavior 5 - suppressed activity, motor deficit, limited reaction to stimuli 10 - self-isolation, lethargy, coordination disturbance 25 - pain vocalization at slight touch, apathy
Inflammation, respiration rate and quality	0 - no saliences 5 - redness/swelling on small body areas 10 - disseminated swellings, labored breathing, low breathing rate 25 - open wounds, gasping, extremely reduced respiration

### X-Ray Micro-Computed Tomography (µCT)

Hind limbs were excised and fixed and were then washed with tap water for 30 min. Thereafter, hind limbs were stored overnight in 154 mM NaCl which acted as the scanning medium. X-ray image acquisition was performed using a voxel size of 9 µm^3^ on a SkyScan 1076 (Bruker, Antwerp, Belgium) with the source operated at 49 kVp and 200 µA. Beam hardening was reduced using a 0.5 mm Aluminum-filter. Rotation steps were set at 0.6°. Frame averaging was performed by 3 repetitions for each projection with an integration time of 1,700 ms. Attenuation coefficient calibration for bone mineral density (BMD) analysis was prepared by associated measurements of two phantoms with densities of 0.25 and 0.75 g/cm^3^, respectively. Image processing was performed in the software pipeline from Bruker as described previously (33). Reconstruction of X-ray images and subdivision of hind limbs into femur, tibia, and paw was conducted with NRecon using a Gaussian filter, smoothing kernel of 2, defect pixel masking of ≤ 20%, ring artifact reduction of 6, beam hardening correction of 30%, and a misalignment compensation depending on image quality. Bones were spatially aligned within the DataViewer software. Selection of volumes and regions of interest were conducted in the CTAn software. For the femur, the proximal reference level was at the fusion site between the greater trochanter and the femoral head. The distal metaphyseal growth plate was set as the second reference point. For analysis of paws, the whole area containing Cuboid, Navicular and Cuneiform bones was analyzed. This region was chosen for the determination of cortical bone parameters and BMD. The cortical bone of the femur was analyzed in the diaphysis and in the epiphyses in proximity to the knee joint. Trabecular bone parameters were also determined at the distal epiphysis. Built-in algorithms were utilized for the determination of parameters of binarized images by 2D plate model for cortical bone (global threshold 64–255) and 3D model for trabecular bone (global threshold 75–255). Three-dimensional reconstructions of knee joints were performed using the CTVol software.

### Histology and Immunohistochemistry

Paws were decalcified using USEDECALC (MEDITE, Burgdorf, Germany). Samples were subsequently dehydrated and embedded into paraffin. Serial sections of 5 µm thickness were then generated from the plantar plane of the paws. For the investigation of immune cell infiltration and tissue deterioration, H&E staining of deparaffinized and rehydrated sections was performed. Verification of bacterial colonization was performed *via* immunohistochemistry. Sections were equilibrated in TBS-T (50 mM TRIS-HCl, pH 7.6, 150 mM NaCl, 0.2% Tween 20). Endogenous peroxidases were inactivated using 3% H_2_O_2_ (Bio-Rad) for 15 min with subsequent rinsing in tap water and TBS-T. Unspecific antibody binding sites were blocked by incubation with 5% normal rabbit serum (Thermo Fisher Scientific, Waltham, MA, USA) for 2 h. After washing with TBS-T, the sections were incubated with 0.5 µg/ml primary polyclonal Goat IgG anti-*Streptococcus pyogenes* Group A carbohydrate (ab9191, abcam, Cambridge, UK) overnight at 4°C. Goat IgG (abcam) was used as an isotype control at the same concentration. On the following day, 0.5 µg/ml secondary Rabbit IgG anti-Goat IgG (Thermo) conjugated with horseradish peroxidase (HRP) was applied for 1 h at room temperature. Subsequently, the sections were stained with the DAB Substrate Kit (Cell Signaling Technology, Danvers, MA, USA) and haematoxylin (Merck Millipore, Burlington, MA, USA). All histological sections were imaged using the Axioplan 2 (Carl Zeiss, Oberkochen, Germany).

### Lipidomics

#### Eicosanoid Extraction

Frozen paw and liver samples were pulverized using a CP02 automated cryoPREP (Covaris, Woburn, MA, USA). The samples were transferred in a Covaris tissue tube, cooled down in liquid nitrogen and then pulverized. From the resulting powder 50 mg was taken, weighed for data normalization and immediately immersed in 500 µl ice cold methanol containing 0.1% butylated hydroxytoluene, 30 nM AUDA (liver samples only) and 500 µl ice cold water. 100 µl of deuterated internal standard mixture was added to each sample. The internal standard consisted of 12-HETE-d_8_, 13-HODE-d_4_, PG E_2_-d_4_, Resolvin D1-d_5_, and AA-d_11_ (Cayman Chemicals, Ann Arbor, MI, USA). For paw samples, an extra lysis step was performed using Fastprep™ (MP Biomedicals, Irvine, CA, USA) with lysing matrix B for 45 s and 6 m/s. For the liver samples, an alkaline hydrolysis step was performed using 300 µl sodium hydroxide (10 M) for 30 min at 60°C. Immediately thereafter, 300 µl sodium acetate solution (1 M) was added on ice and the pH was adjusted for both paw and liver samples to 6 using 10 M acetic acid. Subsequently, solid phase extraction (SPE) was performed using Agilent (Santa Clara, CA, USA) SPE cartridges Bond Elut Certify II. SPE cartridges were conditioned with 3 ml methanol and then with 3 ml 0.1 sodium acetate buffer at pH 6 containing 5% methanol. The samples were loaded and washed with 3 ml of 50% methanol. Eicosanoids were eluted using 2 ml of hexane/ethyl acetate (75/25) containing 0.1 M acetic acid.

#### Eicosanoid Measurement

Extracts were dried under nitrogen flow (TurboVap^®^ from Biotage, Uppsala, Sweden) and reconstituted in 70 µl 80% acetonitrile for liver or 70 µl 25% acetonitrile for paw samples. Dynamic multiple reaction monitoring LC-MS/MS analysis was performed using an Agilent HPLC system (1200 series), coupled to an Agilent 6460 Triple quadrupole mass spectrometer with electrospray ionization source in negative mode. The separation was done with a Gemini^®^ (Phenomenex, Torrance, CA, USA) NX-C18 column (3 µm, 100 × 2 mm) and equivalent pre-column. Calibration curves with MS-certified standards for absolute quantification (range between 0.5 ng/ml and 50 ng/ml for HETEs and EETs; for prostaglandins 0.25 ng/ml to 50 ng/ml and 5 ng/ml to 1,000 ng/ml for precursors, curve type quadratic, weighting 1/x) and deuterated internal standard were used. Eicosanoids classes without appropriate MS-certified standards (HEPE, HODE; HDHA) were normalized to the response of the internal standard and stated in arbitrary units (AU) in the plots. Agilent Mass Hunter Qualitative Analysis software and Agilent Mass Hunter Quantitative Analysis software (both version B.07.00) were used for MS data analysis.

### Serology

For the determination of mouse IgG anti-GAS titers, an in-house enzyme linked immunosorbent assay (ELISA) was performed. 1 ml bacteria suspension (~ 10^8^ CFU/ml) in 6 Well plates was irradiated for 5 min in a GS Gene Linker UV Chamber (Bio-Rad). Complete inactivation of GAS was confirmed using blood agar plates. Suspensions were centrifuged at 4,000 × g for 10 min. Pellets were suspended in 50 mM CO_3_^2-^/HCO_3_^-^ buffer (pH 9.4) and 100 µl of this suspension containing 10^7^ inactivated bacteria was coated onto Nunc MaxiSorp plates (Thermo Fisher Scientific) overnight at 4°C. After washing with PBS-T (DPBS, 0.05% Tween-20), 200 µl blocking solution (DPBS, 2% BSA) was applied for 1 h at room temperature. Plasma samples were diluted 1:800 and subsequently incubated for 1.5 h at room temperature. 100 µl Rabbit F(ab’)_2_ anti-mouse IgG : HRP detection antibody (Bio-Rad) was then applied at a concentration of 0.5 µg/ml. For color reaction, 100 µl of the TMB Substrate Set was used (BioLegend, San Diego, CA, USA) and incubated for 10 min. Enzyme activity was then quenched using 100 µl H_2_SO_4_ (0.5 M). Absorbance signals at 450 nm were obtained by the automated plate reader HT3 (Anthos Mikrosysteme, Krefeld, Germany). For the determination of cytokine concentrations in plasma samples, the LEGENDplex Mouse Inflammation panel (BioLegend) was used containing capture beads and detection antibodies for Interleukin (IL-)1α, IL-1β, IL-6, IL-10, IL-12p70, IL-17A, IL-23, MCP-1, IFNβ, IFNγ, TNFα, and GM-CSF. The protocol was followed using the manufacturer’s instructions. Data acquisition was performed using a FACSVerse (Becton Dickinson).

### Osteoclastogenesis

Bone marrow cells were obtained as described (34). In short, mouse hind and fore limbs were excised and soft tissue was completely removed. Bone marrow cells were acquired by centrifuging long bones at 10,000 × g for 15 s in a perforated 0.5 ml tube nested in a 1.5 ml tube. Cells were counted and then suspended in αMEM with 10% FCS and 1% Penicillin/Streptomycin (Thermo Fisher Scientific). Bone marrow cells were seeded into 6 or 12 well culture plates at a density of 2 × 10^5^ cells/cm^2^ and incubated with 30 ng/ml M-CSF (R&D Systems, Minneapolis, MN, USA) for 24 h at 37°C and 5% CO_2_. Cells were then stimulated using 50 ng/ml RANKL (R&D Systems) along with 100 ng/ml of the TLR2 agonistic lipopeptide Pam2CSK4 (InvivoGen, San Diego, CA, USA) and UV-inactivated GAS, respectively. For coculture experiments, osteoblasts were isolated as described previously (35). In brief, 2-3 days old mice were sacrificed by decapitation. After rinsing the heads with 70% Ethanol and DPBS, Calvaria were excised and cut into 4 mm^2^ pieces. After washing thrice with DPBS, calvaria fragments were immersed in 4 ml digestion solution containing 280 U/ml collagenase type II and 0.25% Trypsin in HBSS (Thermo Fisher Scientific). Bones were subsequently incubated at 37°C for 20 min with vigorous shaking in-between. Bone cells containing supernatants were then separated from the calvaria and the enzyme reaction was quenched by adding 700 µl FCS. The digestion was repeated three times and all supernatants were pooled thereafter. Calvaria cells were seeded into cell culture plates at a density of 0.2 × 10^5^ cells/cm^2^ and were cultured for 24 h. Afterwards, bone marrow cells isolated from long bones as described above were added at a density of 3.8 × 10^5^ cells/cm^2^. Then, the coculture was incubated for 48 h until stimulation with 10 nm 1α,25-Dihydroxyviatmin (D_3_, Sigma-Aldrich, St. Louis, MO, USA) and 1 µM PGE_2_ (Cayman Chemicals) with or without UV-inactivated GAS and Pam2CSK, respectively. Culture was continued for 10 days while adding D_3_ and PGE_2_ every 24 h. Multinucleated osteoclasts were identified using the tartrate resistant acid phosphatase (TRAcP) kit (Sigma-Aldrich).

### Fibroblast-Like Synoviocytes Culture and Infection

Fibroblast-like synoviocytes (FLS) were extracted from murine paws. In brief, mice were sacrificed and rinsed with 70% Ethanol. Hind and forelimbs were removed and put into cold FLS-Medium composed of DMEM, 10% FCS, 1% Penicillin/Streptomycin, 2 mM L-Glutamine (Thermo Fisher Scientific), 10 mM HEPES, and 1 mM pyruvate (PAN-Biotech, Aidenbach, Germany). The following steps were conducted under sterile conditions. The skin was removed and the tendons of the digits were transected. After removal of any soft tissue, paws were isolated by overflexing ankle and wrist joints, respectively. Afterwards, paws from each animal were individually immersed in 240 U/ml collagenase type IV (STEMCELL Technologies, Vancouver, Canada) and digested for 1 h at 37°C with gentle agitation. Organ debris was then removed using a 70 µm cell strainer (Greiner Bio-One, Kremsmünster, Austria) and cells were suspended in FLS-Medium. After quantification of cell numbers, 10^6^ FLS were cultured in T75 cell culture flasks (TPP, Trasadingen, Switzerland) until full confluency. Purification of cell cultures was achieved by using the CD45 S-pluriBead (pluriSelect, Leipzig, Germany) separation technique after the manufacturer’s instructions. Purity of FLS was verified using a characterization panel consisting of anti-CD106:PerCP-Cy5.5 (clone 429), anti-CD11b:PE (M1/70), anti-CD31:APC (390), anti-CD45:FITC (30-F11), anti-CD54:APC/Fire750 (YN1/1.7.4), anti-CD90.2:BV605 (30-H12), anti-Gr-1:PE-Cy7 (RB-8C5), and DAPI for discrimination of dead cells (all from BioLegend). Cells were counted and suspended in autoMACS Running Buffer (Miltenyi Biotec, Bergisch Gladbach, Germany). TruStain FcX (BioLegend) was used for blocking CD16 and CD32 by incubation for 10 min on ice. The antibody cocktail was subsequently applied for 20 min at room temperature. After washing and suspending in 200 µl Running Buffer, cells were analyzed on a FACSAria IIIu utilizing the FACSDiva Software v8.0.2 (Becton Dickinson). All CD31^−^CD45^−^ cells were considered FLS. Flow cytometry data was analyzed using the FlowJo software (Becton Dickinson). Purified FLS were then again cultured until full confluency was reached and after washing thrice with DPBS, 3 × 10^5^ cells in 2 ml αMEM (Thermo) with 10% FCS were subsequently seeded into 6 well plates. On the following day cells were infected with 3 × 10^6^ bacteria (≈ MOI 10) for 4, 8, or 12 h. Non-infected cells served as a control along with cells incubated with UV-inactivated bacteria. For subsequent transcription analyses, cells were immersed in 700 µl Buffer RLT (Qiagen, Hilden, Germany) and lyzed using a cell scraper and vigorous vortexing. Suspensions were then snap frozen and stored at -80°C. Preparation for flow cytometry included washing of the cells with warm DPBS and digestion with trypsin at 37°C for 1 min. After quenching with medium, cells were suspended in autoMACS running buffer and put on ice until labeling as described above.

### Gene Expression Analysis

RNA was isolated using the RNeasy Plus Mini Kit (Qiagen) to the manufacturer’s instructions. RNA quantities were determined spectrophotometrically using the NanoDrop ND1000 (Thermo Fisher Scientific) and 500 ng were submitted to cDNA synthesis utilizing the High-capacity cDNA Reverse Transcription Kit (Applied Biosystems, Foster City, CA, USA). For qPCR analysis TaqMan primer pairs and probes were used for *Ccl2* (Assay ID: Mm00441242_m1), *Cxcl2* (Mm00436450_m1), *Il1b* (Mm00434228_m1), *Il6* (Mm00446190_m1), *Il10 (*Mm00439614_m1*)*, *Mmp13* (Mm00439491_m1), *Opg* (Mm01205928_m1), *Rank* (Mm00437132_m1), *Rankl* (Mm00441906_m1), *Tnf* (Mm00443258_m1), and *Gapdh* (Mm05724508_g1) as a reference gene (Applied Biosystems). All reactions were amplified using TaqMan Gene expression Master Mix and analyzed on the Applied Biosystems 7900 Fast Real-Time PCR System.

### Statistical Analysis

Data visualization and statistics were performed using R (v3.6.3). For survival and probability of incidence analyses, groups were compared using the logrank test. Bivariate correlation was evaluated using the Pearson product-moment correlation coefficient (*R*). Variables with *R* ≥ 0.6 were considered to correlate strongly. Univariate statistical analyses were performed under the assumption of non-normally distributed data. Medians were visualized using boxplots depicting interquartile ranges (IQR) within the box and whiskers for values within 1.5 × IQR. Comparisons of multiple groups were conducted using the Kruskal-Wallis test combined with the post-hoc Mann-Whitney U test for pairwise comparison including adjustments of p values with the Bonferroni-Holm method. When comparing medians to a standard value, the one-sample Wilcoxon signed rank test was performed. For sample sizes of n = 3 the Student’s t-test was applied. P values of ≤ 0.05 were considered statistically significant. Significance levels are abbreviated as follows: *p < 0.05, **p < 0.01, ***p < 0.001.

## Results

### GAS Infection Led to Sepsis and Septic Arthritis Characterized by Bacterial Colonization and Neutrophil Infiltration Into the Joints

**Figure 1:**
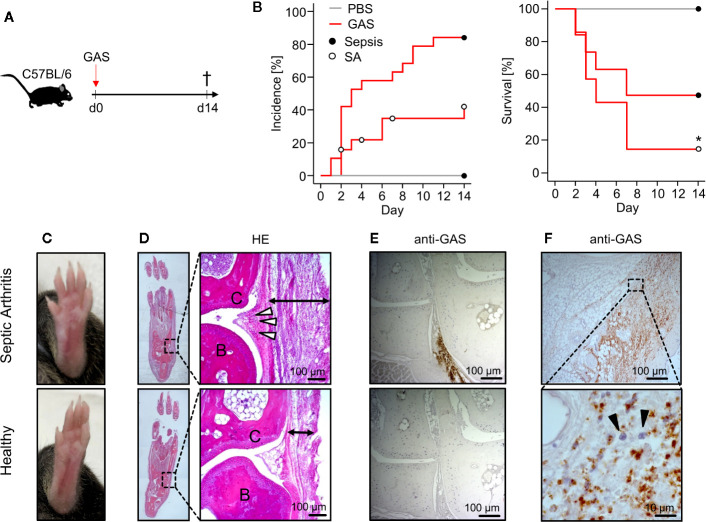
Mice infected with Group A Streptococcus (GAS) show signs of rapidly progressing septic arthritis (SA) including immune cell infiltration and bacterial colonization of joint areas. **(A)** Experimental scheme for the infection of 20-22 week old male C57BL/6 mice with 5 ˣ 10^5^ CFU of GAS (n = 19). **(B)** Left panel: Kaplan-Meier estimator of incidence functions for sepsis and SA of infected animals (n = 19) and PBS controls (n = 4). Right panel: Survival rates of mice not affected by SA (n = 12) compared to mice with SA (n = 7). Open and closed circles denominate censored data, respectively. **(C)** Representative pictures showing swollen paw (top) after GAS infection in contrast to healthy paw (bottom). **(D)** Hematoxylin and eosin (HE)-stained sections in the frontal plane of the paws showing that the infection-related swelling of paws is a result of immune cell migration into the subcutaneous (black arrows) and periarticular soft tissue (white arrowheads). Upper panel: paw affected by septic arthritis. Lower panel: healthy paw. B: bone, C: cartilage. **(E, F)** Immunohistochemistry using the anti-Group A Carbohydrate antibody revealing the bacterial colonization in paw sections. **(E)** Areas around transverse tarsal joints are shown. Upper panel: goat anti-Group A Carbohydrate. Lower Panel: goat IgG isotype control. (**F**) Bacterial colonization of subcutaneous tissue accompanied by neutrophil infiltration (black arrowheads) identified by their nuclei’s morphology in the hematoxylin staining. Images of histological sections are representative for mice with macroscopic swellings (n = 5).

We first explored the Group A Streptococcus (GAS) strain AP1 for its capacity to induce sepsis after intravenous infection in C57BL/6 mice (**Figure 1A**). Sepsis was scored *via* assessment of clinical signs of burden as summarized in **Table 1**. Accordingly, macroscopic signs of animal stress that resulted in a sepsis score ≥ 5 were considered sepsis occurrence (*Materials and Methods*). Under this scoring, over 80% (16/19) of mice infected with 5 × 10^5^ colony-forming units (CFU) developed sepsis (**Figure 1B**, left panel) and about 40% (7/19) exhibited a distinct swelling of paws (**Figure 1C**). Of note, the development of swollen limbs was accompanied by a considerable decrease of survival rate to below 20% when compared to animals without macroscopic signs of arthritis (p = 0.04, **Figure 1B**, right panel). Surprisingly, infection with 4 × 10^5^ CFU resulted in less than 20% of mice developing septic arthritis and a 90% survival rate. Reducing the infectious dose further to 1 × 10^5^ CFU neither induced septic arthritis nor mortality (**Figure S1A**). **Figure S1B** compares the sepsis scores resulting from the various infectious doses and **Figure S1C** confirms for the survivors of sepsis an immune response characterized by the production of GAS specific IgG. Altough the bacterial burden in the blood was increased at 5 × 10^5^ CFU, it did not reach statistical significance when compared to lower doses of infection due to high a variance in the data. Also, the bacterial counts in liver and spleen did not change with varying doses of pathogens (**Figure S1D**).

Hematoxylin-Eosin stained thin sections of swollen paws revealed cellular infiltrates in the periarticular soft tissue surrounding the tarsal joints (**Figure 1D**). In order to prove the presence of GAS *in situ*, we established an immunohistochemistry assay using anti-Group A Carbohydrate antibodies and indeed detected bacteria directly within the joint microenvironment (**Figure 1E**). As we predicted that bacterial invasion into the joints would attract immune cells, we were interested in the particular type of cell and identified neutrophilic granulocytes - recognized by their segmented nuclei - co-localizing with GAS (**Figure 1F**). Taken together, we here established an *in vivo* model of sepsis that led to septic arthritis characterized by bacterial invasion and neutrophil infiltration into the joints.

### GAS Infection Was Associated With Increased Eicosanoid Levels in the Paws

**Figure 2:**
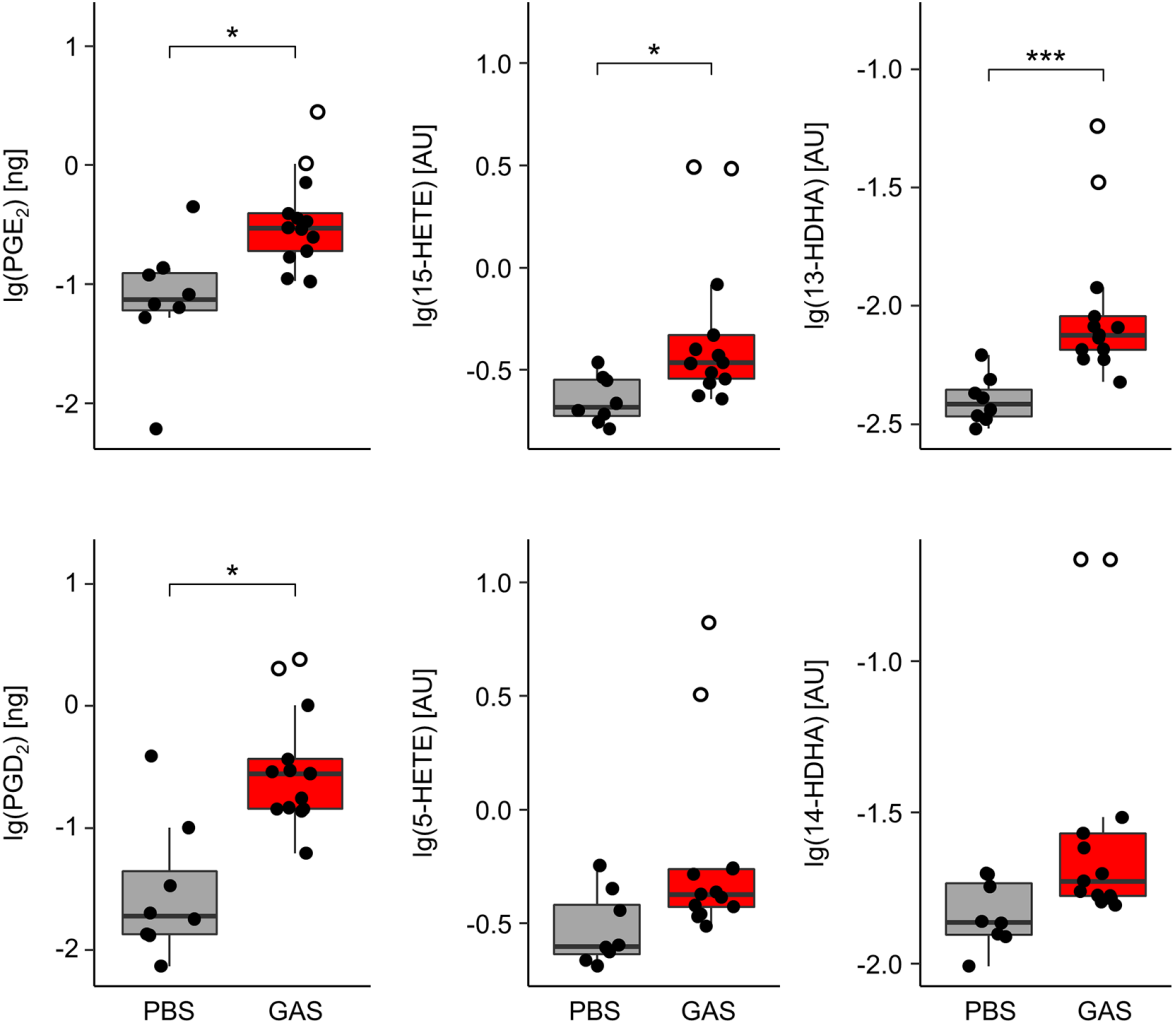
Group A Streptococcus (GAS) infection stimulates the overproduction of paw eicosanoids in infected mice. Paws from C57BL/6J mice were homogenized and eicosanoids were extracted for lipidomics analyses by HLPC-MS/MS using dynamic multiple reaction monitoring. Samples from infected animals (n = 13) showed significantly elevated levels of prostaglandins (PG) E_2_, D_2_, 15-hydroxyeicosatrienoic acid (15-HETE), and 13-hydroxydocosahexaenoic acid (13-HDHA) when compared to samples from uninfected controls (n = 8). Open circles represent paws with septic arthritis. lg: common logarithm, AU, arbitrary units. *p < 0.05, ***p < 0.001, Mann-Whitney U test.

In order to monitor eicosanoids as prime mediators of immune activation and resolution, we employed HPLC-MS/MS on lipid extracts of the paws from C57BL/6J mice. We could thus show that GAS infection led to a marked elevation of the pro-inflammatory prostaglandins (PG) E_2_ and D_2_ (**Figure 2**) resulting from cyclooxygenase activity. Likewise, GAS induced a significant increase in lipoxygenase catalyzed conversion to hydroxyeicosatrienoic acids (HETEs) and in particular 15-HETE, while the increase in 5-HETE did not quite reach statistical significance. Lastly, GAS also led to an increase in 13- and 14-HDHA derived from docosahexaenoic acid (DHA). Of note, the outliers of our data correspond to paws showing macroscopic signs of arthritis (**Figure 2**). In summary, we here showed for the first time that GAS infection resulted in a significant increase of eicosanoids in the paws.

### UV-Inactivated GAS Inhibited Osteoclastogensis *In Vitro*

In order to monitor whether GAS infection, sepsis or septic arthritis impacted on bone morphometry, we employed micro-computed tomography (µCT). To that extent, we compared PBS treated animals to those infected. While all of the control animals reached experimental day 14 as endpoint, 47% of the infected mice had to be sacrificed between experimental days 2 and 4, due to high sepsis scores. To avoid a selection bias in our data, samples from all time points were used and combined into one GAS group. μCT analysis was performed on femora and paws. As for the femora, we concentrated on the distal epiphysis and differentiated trabecular from cortical bone while for the paws, we analyzed the cortical bone of the tarsus, only. As shown in **Figure S2**, we did not detect any alterations to the bone morphometry associated with GAS infection.

As the time frame for developing *in vivo* bone erosions may have been too short, we went on to investigate if GAS had any potential to manipulate bone homeostasis by impacting on the balance of bone resorbing osteoclasts and bone synthesizing osteoblasts. To that extent, we set up *in vitro* experiments and analyzed how GAS influenced on osteoclastogenesis. For that, we stimulated murine bone marrow cells with RANKL - a cytokine essential for osteoclast development - and UV-inactivated bacteria (UV-GAS) and subsequently identified multinucleated Osteoclasts (OCs) *via* TRAcP-staining, which is selective for the target cells (**Figure S3A**). Indeed, the application of UV-GAS dose-dependently led to a significant reduction in OC counts and resulted in mononucleated yet TRAcP-positive progenitors, only. In contrast, simple TLR2 activation *via* its agonist Pam2CSK4 significantly promoted OC differentiation (**Figures S3B, C**). As expected, the application of M-CSF on BM cultures without RANKL stimulation did not yield any TRAcP-positve precursor cells (**Figure S3B**).

To address the cross-talk between osteoblasts and osteoclasts, we established an *in vitro* co-culture model of calvaria-derived osteoblastogenic cells and mononuclear cells from the bone marrow. RANKL production by osteoblastogenic cells was initiated by stimulating with a bioactive Vitamin D_3_ derivate and PGE_2_ (**Figure S4A**). Under these conditions, multinucleated and TRAcP-positive osteoclasts developed and again, stimulation with the TLR2 agonist Pam2CSK4 enhanced this development. However, as was the case for single osteoclast cultures, UV-GAS inhibited osteoclast development (**Figure S4B**). Gene-expression analyses of these co-cultures yielded decreased expression levels of *Rankl*, *Rank*, and *Opg* as well as *Tnf*, *Il1b*, and *Il10* in the presence of UV-GAS (**Figure S4C**). In summary we did not observe any changes to the bone mass following GAS infection *in vivo*, even if GAS can perturb the RANK-RANKL-OPG axis and prevent osteoclastogenesis *in vitro*.

### GAS-Infected FLS Upregulated Mediators of Immune Cell Attraction and Retention

**Figure 3:**
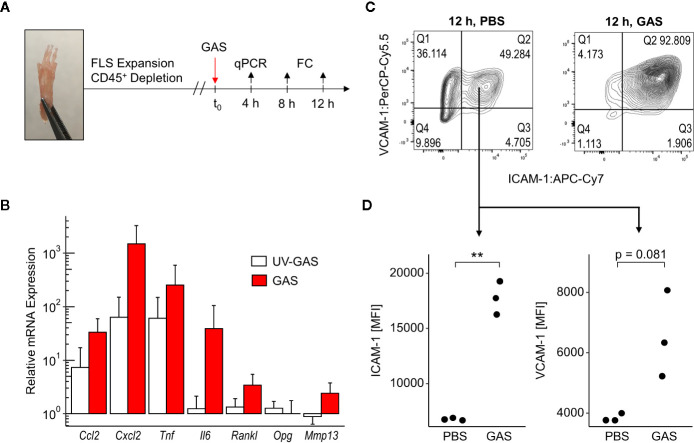
Fibroblast-like synoviocytes (FLS) are triggered after Group A Streptococcus (GAS) infection to upregulate chemokines, pro-inflammatory cytokines and integrin ligands. **(A)** Experimental design of the *in vitro* infection model. FLS were isolated from murine paws by digestion with collagenase type IV and were purified by depleting leukocytes. GAS was applied at a multiplication of infection of 10. FC: flow cytometry. **(B)** Relative mRNA expression levels measured by quantitative PCR 4 h after GAS infection. Ct values were normalized to uninfected FLS and the housekeeping gene *Gapdh* by the 2^-(ΔΔCt)^-Method. Means + SD are depicted in bar plots. The bar graph shows gene-expression levels from infected cells (n = 4) and FLS stimulated with an equivalent number of UV-inactivated bacteria (UV-GAS, n = 3). **(C)** Representative contour plots of flow cytometry data 12 h into the infection experiment depicting subpopulations of CD31^−^CD45^−^ FLS. GAS infection promotes the shift to ICAM-1^+^VCAM-1^+^ fibroblasts. **(D)** Expression of ICAM-1 and VCAM-1 represented by mean fluorescence intensities (MFI) of CD31^−^CD45^−^ICAM-1^+^VCAM-1^+^ FLS. **p < 0.01, Mann-Whitney U test.

To further address how GAS led to septic arthritis, we investigated the contribution of the synovial membrane to the disease process. To that extend, we isolated fibroblast-like synoviocytes (FLS) - the dominant cell type of the synovial membrane - and infected them *in vitro* with GAS at a multiplication of infection (moi) of 10 for 4, 8 or 12 h (**Figure 3A**). Transcription analyses at 4 h post infection revealed an over 30- and 1,000-fold increment in the transcription of the chemokine genes *Ccl2* and *Cxcl2*, respectively (**Figure 3B**). Likewise, the expression of the genes encoding pro-inflammatory cytokines *Il6* and *Tnf* were increased 40- and 200-fold, respectively. UV-inactivated GAS on the other hand was also capable of inducing an upregulation of *Ccl2*, *Cxcl2* and *Tnf* however, to a lesser extent than live bacteria and had only a negligible impact on *Il6* transcription. Infection with live GAS also promoted a 3-fold increase in *Rankl* expression whereas the level of *Opg*, whose protein product opposes the action of RANKL, remained apparently unaffected. Likewise, live GAS also augmented the transcription of the gene encoding for the matrix metalloprotease *Mmp13*. Levels of secreted proteins were almost consistently confirmed for CCL2, CXCL2, TNF, and IL-6. These cytokines were time-dependently increased in supernatants from infected cultures with the exception of CXCL2 at 8 h and IL-6 at 24 h p.i., respectively, due to low sample sizes and high variances (**Figure S5**). The capacity of FLS to adjust the expression of integrin ligands in response to GAS infection was assessed *via* flow cytometric analyses (**Figure S6**). Within 12 h of infection, we observed an almost complete shift of the CD31^−^CD45^−^ FLS population towards ICAM-1^+^VCAM-1^+^ cells (**Figure 3C**), indicating that more than 90% of all FLS expressed these adhesion molecules at a high level (**Figures 3C, D**). Together, these results suggest that FLS are involved in the initiation and perpetuation of an immune response against GAS. Furthermore, they are possibly able to modulate bone remodeling by increasing *Rankl* production while maintaining *Opg* expression consistent, which would benefit amplified osteoclastogenesis.

### Pre-Existing Inflammatory Joint Disease Exacerbated Sepsis and Promoted the Incidence of Septic Arthritis

**Figure 4:**
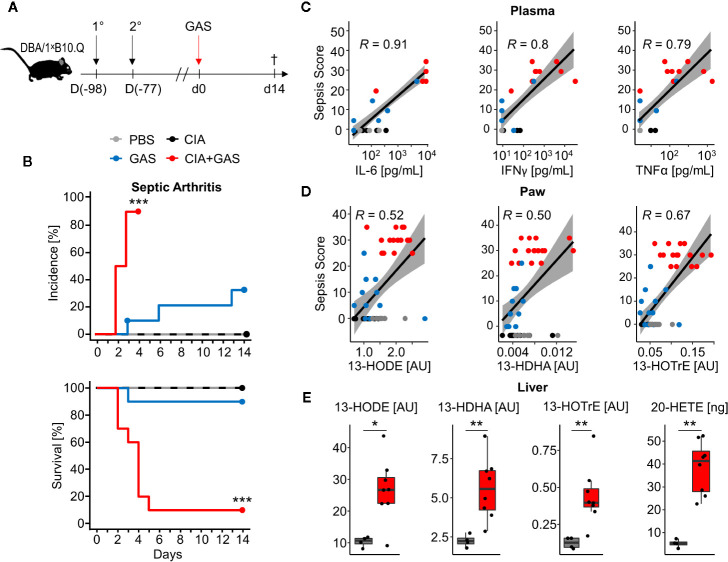
Preceding collagen-induced arthritis (CIA) aggravates sepsis and septic arthritis. **(A)** Scheme for experimental procedure. 6-8 week old DBA/1 ˣ B10.Q mice of the F1 generation were immunized with bovine collagen type II in complete Freund’s adjuvant (1°) and were boosted 3 weeks later (2°). CIA developed and was monitored for 11 weeks prior to infection with 2 ˣ 10^6^ colony-forming units of Group A Streptococcus (GAS). **(B)** Kaplan-Meier estimator curves of septic arthritis incidence (top) and survival (bottom) illustrate that preceding CIA (n = 10) exacerbates disease severity which is accompanied by a significant increase of septic arthritis occurrence when compared to mice without a previous CIA (n = 10). ***p < 0.001, logrank test. **(C, D)** Bivariate linear correlation of sepsis scores with plasma levels of pro-inflammatory cytokines in plasma **(C)** and amounts of eicosanoids in paws **(D)**. Mice with combined CIA and GAS infection show the highest sepsis scores which moderately to strongly correlate with Interleukin (IL-)6, Interferon (IFN)γ, Tumor Necrosis Factor (TNF)α, 13-Hydroxyoctadecadienoic acid (13-HODE), 13-Hydrodxydocosahexaenoic acid (13-HDHA) and 13-Hydroxyoctadecatrienoic acid (13-HOTrE). Gray areas depict the 0.95 confidence interval of the regression line. *R*: Pearson product-moment correlation coefficient. **(E)** Boxplots showing the increased eicosanoid levels in liver from mouse with CIA and GAS infection (red boxes, n = 8) compared to PBS controls (gray boxes, n = 4). *p < 0.05, **p < 0.01, Mann-Whitney U test.

To investigate whether chronic inflammatory joint disease promoted the incidence and severity of SA, we combined GAS infection with collagen-induced arthritis (CIA). To that extent, CIA was induced in genetically susceptible DBA/1 × B10.Q (F1) mice by primary and secondary immunization with bovine collagen type II (**Figure 4A**). Thereafter, the development of CIA was monitored until the start of the remission phase, 11 weeks post immunization, when mice were infected by GAS. By the time of the infection, the swelling of paws induced by CIA had subsided, hence allowing subsequent detection of SA signs (**Figure S7**). When comparing naïve controls to CIA mice, the incidence of SA significantly increased from 30 to 90% (**Figure 4B**). Moreover, SA developed in paws formerly affected by CIA but was not restricted to these sites. Of note, higher numbers of bacteria were isolated from the knee joints of mice with pre-existing CIA and likewise, bacteremia was facilitated as demonstrated by increased bacterial burdens in the blood, liver and spleen (**Table 2**). Thus, sepsis was exacerbated in CIA mice, reducing the survival rate to 10% within 5 days after infection (**Figure 4B**).

**Table 2:** Pre-existing collagen induced arthritis (CIA) promoted bacteremia and colonization of tibiofemoral joints.

	Median bacterial burden (IQR) [CFU/ml ˣ 10^5^]		
	GAS	CIA+GAS	p
Knee (left)	<0.005 (0.005 – 0.005)	0.010 (0.005 – 0.52)	0.036*
Knee (right)	<0.005 (0.005 – 0.005)	0.015 (0.005 – 0.18)	0.058
Blood	<0.001 (0.001 – 0.001)	10 (0.51 – 10)	0.012*
Liver	<0.002 (0.002 – 0.002)	532 (38 – 800)	0.0039**
Spleen	<0.001 (0.001 – 0.001)	194 (2 – 800)	0.0039**

GAS were isolated, selected and counted on blood agar. p values depict significance levels when comparing bacterial counts of the CIA+GAS group with the respective limits of detection. *p < 0.05, ** p < 0.01, one-sample Wilcoxox signed rank test.

To further investigate if exacerbated sepsis was paralleled by a cytokine storm, we analyzed plasma cytokines using a multiplexed flow cytometry approach. Sepsis scores were thus found to be in strong linear correlation with the concentrations of IL-6, IFNγ, and TNFα and again, higher sepsis scores in mice with pre-existing CIA correlated with elevated levels of these cytokines (**Figure 4C**). Notably, mice suffering from CIA only did not display elevated levels of pro-inflammatory cytokines and were comparable to PBS controls and mice infected with GAS only (**Figure S8**).

Lipidomic analyses of the paws demonstrated correlations of the anti-inflammatory lipid mediators 13-HODE, 13-HDHA, and 13-HOTrE with the respective sepsis scores. As was the case for cytokines, CIA itself did not cause an increase in eicosanoid levels (**Figure 4D**). Moreover, eicosanoid levels in paws correlated positively with systemic IL-6 concentrations, suggesting interdependencies between immunologically relevant mediators during aggravated sepsis (**Figure S9**). Analysis of the lipid profiles in the liver again confirmed an increase in 13-HODE, 13-HDHA and 13-HOTrE together with pro-inflammatory 20-HETE in mice that were subjected to CIA and GAS infection when compared to PBS controls (**Figure 4E**). In summary, we demonstrated that pre-existing inflammatory joint disease promoted the incidence and severity of SA and increased sepsis scores were paralleled by elevated cytokines and eicosanoids.

### Arthritis Scores Determine Bone Erosion in Septic Arthritis

**Figure 5:**
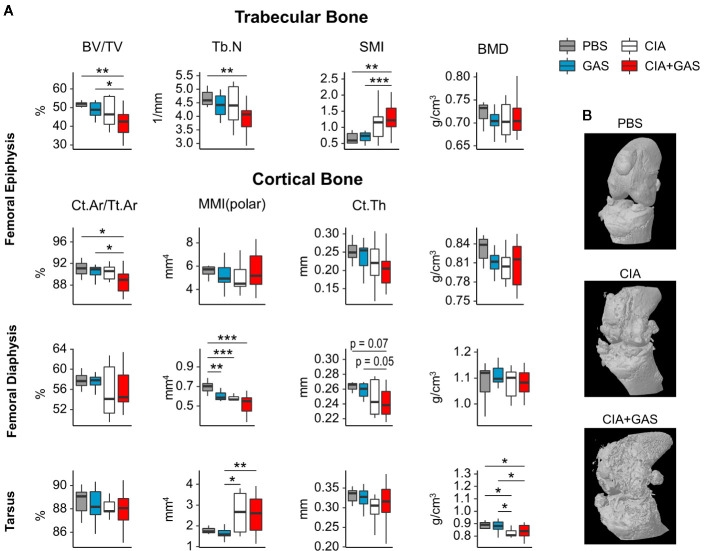
Deterioration of bone morphology due to collagen induced arthritis (CIA) becomes significant when combining the chronic inflammatory joint disease with Group A Streptococcus (GAS) infection. **(A)** Femora and paws were morphometrically assessed by micro-computed tomography. A panel of bone morphology parameters is shown for trabecular bone in the femoral epiphysis and cortical bone in the femur and the tarsus, respectively. Bone volume fraction (BV/TV), structure model index (SMI) and trabecular number (Tb.N) of trabecular bone are affected in the CIA+GAS group. In this Group cortical area fraction (Ct.Ar/Tt.Ar), polar moment of inertia (MMI), cortical thickness (Ct.Th) and bone mineral density (BMD) of certain areas of cortical bone are also modified. **(B)** Representative 3-dimensional reconstructions of the knee joint areas show bone erosion caused by CIA and CIA+GAS, respectively, as a result of detrimentally changed morphology parameters. *p < 0.05, **p < 0.01, ***p < 0.001, Kruskal-Wallis test combined with posthoc Mann-Whitney U test and type I error rate corrections with the Bonferroni-Holm method.

**Figure 6:**
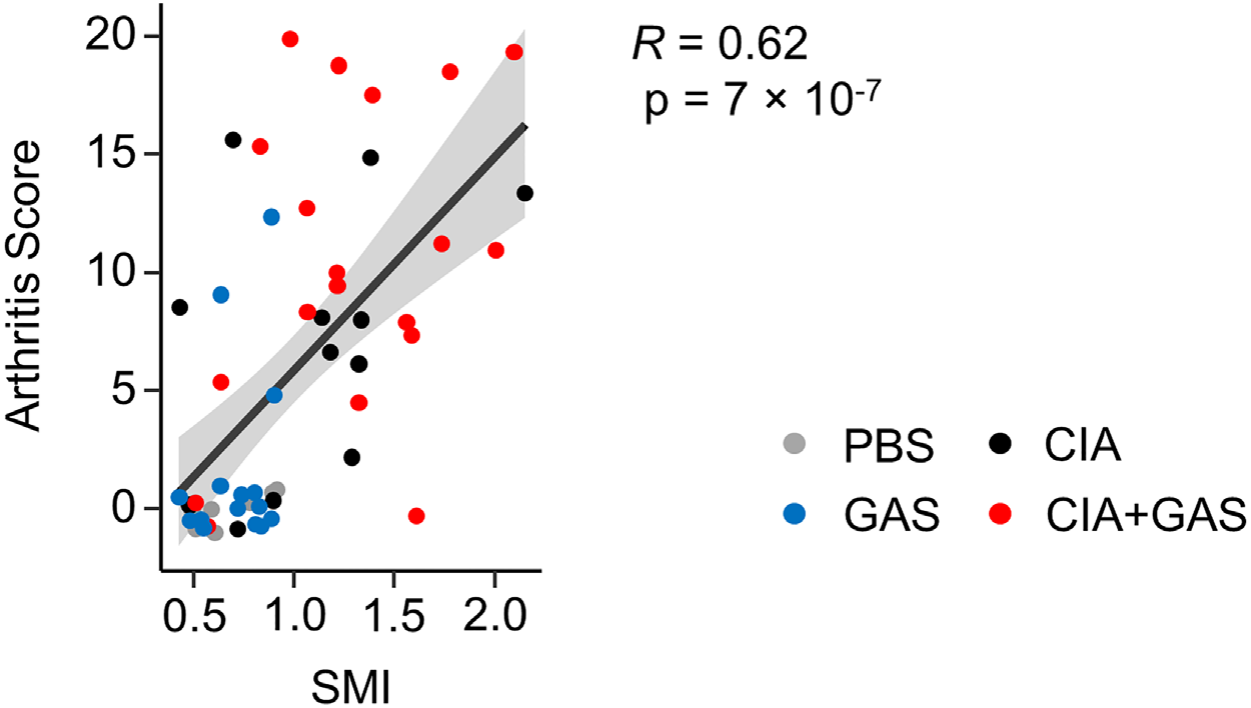
Elevated Arthritis Scores are associated with an osteoporotic phenotype. Dot plot depicting the bivariate correlation analysis of Arthritis Scores and the Structure Model Index (SMI) of the femoral epiphysis. The gray area shows the 0.95 confidence interval of the regression line. *R*: Pearson product-moment correlation coefficient.

In order to investigate if exacerbation of sepsis and SA following GAS infection in CIA mice also involved bone erosion, we here performed µCT analysis and evaluated the resulting bone morphometric data. To that extent, we concentrated on the distal femoral epiphysis for assessment of trabecular bone parameters and found a significant reduction in the bone volume fraction (BV/TV: bone volume [BV] divided by tissue volume [TV]) in CIA+GAS mice compared to PBS controls (**Figure 5A**). This reduction in BV/TV was paralleled by a significant reduction of trabecular numbers (Tb.N). Likewise, there was a significant increase in the structure model index (SMI), which is a measure for trabecular geometry, confirming a more osteoporotic phenotype. Cortical bone parameters were assessed again at the distal femoral epiphysis, the femoral diaphysis and at the tarsus. We observed a decreased cortical area fraction (Ct.Ar/Tt.Ar) - which is defined as the quotient of the cortical bone area and the total cross-sectional area inside the periosteal envelope - close to the knee joints of the CIA+GAS mice. In fulminant cases the deleterious changes in morphology parameters cumulatively led to a grossly deteriorated bone structure of the tibiofemoral joints (**Figure 5B**). However, the cortical bone in the femoral diaphysis was also most affected in the CIA+GAS compared to the PBS control mice. This was shown by reduced cortical thickness (Ct.Th) and polar moment of inertia (MMI) (**Figure 5A**). The latter describes the resistance of the bone to torsional deformation. As for the cortical bone of the tarsus, CIA mice showed comparable parameters to the CIA+GAS mice that were characterized by an increase in the MMI and a reduction in the bone mineral density (BMD). To assess if the severity of joint inflammation determined the magnitude of bone erosion, we correlated arthritis scores to bone parameters. Indeed, **Figure 6** shows a representative correlation analysis between arthritis scores and the corresponding structure model index (SMI). **Table 3** summarizes further correlations. Of note, when analyzing plasma samples from infected mice, concentrations of soluble RANKL where not altered between groups, suggesting that bone erosion was possibly mediated by membrane-bound RANKL (data not shown). In summary, the severity of on-going arthritis determined the magnitude of bone erosions resulting from GAS infection, especially in CIA mice.

**Table 3:** Arthritis scores determine bone erosion in septic arthritis.

Location	Parameter	Arthritis Score	
		*R*	p
Femural Epiphysis	BV/TV	-0.47	***0.00033
	Tb.N	-0.34	*0.013
	BMD	-0.32	*0.017
	Ct.Ar/Tt.Ar	-0.37	**0.005
	MMI(polar)	0.15	0.29
	Ct.Th	-0.33	*0.016
	BMD	-0.40	**0.0026
Femural Diaphysis	Ct.Ar/Tt.Ar	-0.45	***0.00071
	MMI(polar)	-0.21	0.14
	Ct.Th	**-0.60**	***2.0 ˣ 10^-6^
	BMD	-0.33	*0.014
Tarsus	Ct.Ar/Tt.Ar	-0.32	*0.017
	MMI(polar)	0.56	***5.8 ˣ 10^-6^
	Ct.Th	-0.26	*0.050
	BMD	-0.49	***0.00012

The bold value depicts a strong correlation. *p < 0.05, **p < 0.01, ***p < 0.001, Pearson product-moment correlation coefficient (R).

## Discussion

We here demonstrated that inflammatory joint disease posed a risk for Streptococcal sepsis and septic arthritis in mice. Pre-existing CIA significantly reduced survival of subsequent GAS infection and significantly increased incidence of SA with both, synovial fibroblasts and immune cells contributing to the disease process.

Our immune histologic analyses of SA revealed neutrophils co-localizing with bacteria in the joints. Although neutrophils are unconditionally indispensable for reducing the bacterial load during infection, they are also suspected to be involved in the pathogenesis of SA (36). They express high densities of pattern recognition receptors and these receptors may be rapidly activated upon encounter with streptococcal components (37–39). Receptor activation leads to the discharge of inflammatory mediators, thereby attracting further cells and feeding into the derailment of the immune response in SA (40, 41). CIA on the other hand is characterized by continuous proliferation and accumulation of synovial fibroblasts in the joints (42, 43). Our *in vitro* data demonstrated that these FLS are stimulated by GAS to release cytokines and upregulate the expression of integrin ligands, thereby attracting immune cells. In summary our data suggest that, in the case of pre-existing CIA, within the pannus, FLS may encounter invading bacteria, activate and upregulate the expression of cytokines that attract/retain innate immune cells. FLS and neutrophils then act in concert and promote inflammation entailing joint deterioration.

Inflammation in SA has been postulated to manipulate the RANKL/RANK/OPG axis and promote the differentiation of bone osteoclasts thereby accelerating bone resorption (44–47). However, the cellular source of RANKL, the master switch of osteoclast differentiation, has not yet been unambiguously identified. A previous *in vitro* study demonstrated that osteoblastogenic bone cells were able to upregulate RANKL upon GAS infection (48). Interestingly, our *in vitro* data demonstrated that GAS infection of bone cells inhibited RANKL-mediated osteoclastogensis rather than promoting it, which supports the notion that bone erosion is a consequence of the host’s reaction to the infection and not a result of the infection itself (12). However, for an infection of bone cells to occur *in vivo*, an active osteomyelitis is required which was not observed in the present experiments.

We suggest that FLS may be the primary cellular source of RANKL during SA for two reasons: (i) our histological analyses suggested that FLS were in direct contact with GAS during SA and (ii) GAS triggered the upregulation of RANKL (*Tnfsf11*) in FLS cultures to some degree. Furthermore, it has been shown that the inflammatory cytokines TNFα and IL-6 support RANKL production in FLS (49–51). We here confirmed that both, TNFα and IL-6 were markedly increased in CIA mice after infection and this was accompanied by severe bone loss within a few days of SA progression. Mice without the preceding joint condition neither displayed such high cytokine levels nor exhibited any apparent changes in bone morphometry. We therefore suggest that rapid onset of excessive inflammation expedited joint destruction. Similarly, Matsui et al. demonstrated that mice expressing human CD46 - which facilitates GAS infection and therefore promotes a disproportionate inflammatory response (52, 53) - were suffering from pronounced bone lesions within three days after infection (54). The bone mass of infected wild type mice, however, did not change significantly when compared to healthy animals. Another argument supporting FLS as a source for RANKL is our observation of bone loss positively correlating with arthritis severity scores. These account for joint swelling, a result of hyperplasia mostly driven by FLS during inflammation. Yet, we did not detect RANKL production and presentation by FLS *in situ*. To test our hypothesis, future studies must therefore investigate the mechanisms of RANKL-induced bone erosion during SA and the contribution of FLS to osteoclastogenisis *in vivo*.

The combination of GAS with pre-existing CIA led to an increase of several lipid mediators in paws and liver. In detail, the oxylipin 13-HOTrE was also found to be augmented in sera samples of patients with psoriatic arthritis (55). Both 15-lipoxygenase metabolites 13-HODE and 13-HOTrE are able to inhibit the NLRP3 inflammasome complex (56) and increased levels of 13-HODE were also discovered in patients suffering from sepsis (57). Furthermore, the lipid mediator 20-HETE is known to be involved in both sepsis and arthritis. This metabolite is able to influence vasoconstriction and vasodilation by release of nitric oxide (58, 59), to induce cardiac protection in sepsis (60) and inhibit synthesis of prostanoids like PG E_2_ (61). We suggest that high levels of 20-HETE in the liver from CIA+GAS animals (fold change > 7) was a result of the combined disorders.

Despite our novel insights into the etiopathogenesis of SA, there are limitations to our study. We used an animal model for a strictly human pathogen, as GAS is primarily adapted to this host to the extent that many of its substantial virulence factors have activities exclusively against human cells and proteins (62, 63). Yet, our mouse model was sufficiently adequate in representing the haematogenous dissemination of the bacteria, which is the main prerequisite for SA occurrence. Accordingly, we not only observed bacterial load in liver and spleen, but also were able to isolate vital GAS from tibiofemoral joints. On the other hand, GAS infection seemed to have exacerbated pre-existing autoimmunity. Although CIA had entered a remission phase by week 11, uninfected CIA mice showed signs of undergoing autoimmune bone-pathology (e.g., in the femoral diaphysis and tarsus) that became more evident in GAS-infected CIA mice. Therefore, our data support the hypothesis that pre-existing autoimmunity promotes SA, which was derived from epidemiological studies on human cohorts. A minor limitation of this study is the selection of a relatively short observation period of 14 days post infection and animals suffering from SA even had to be sacrificed within a few days of disease progression. Human SA, however, may transit to a chronic disease state with irreversible joint damage and disability in surviving patients (14). We therefore are unable to assess any impact of an adaptive immune response on the perpetuation of joint inflammation.

**Figure 7:**
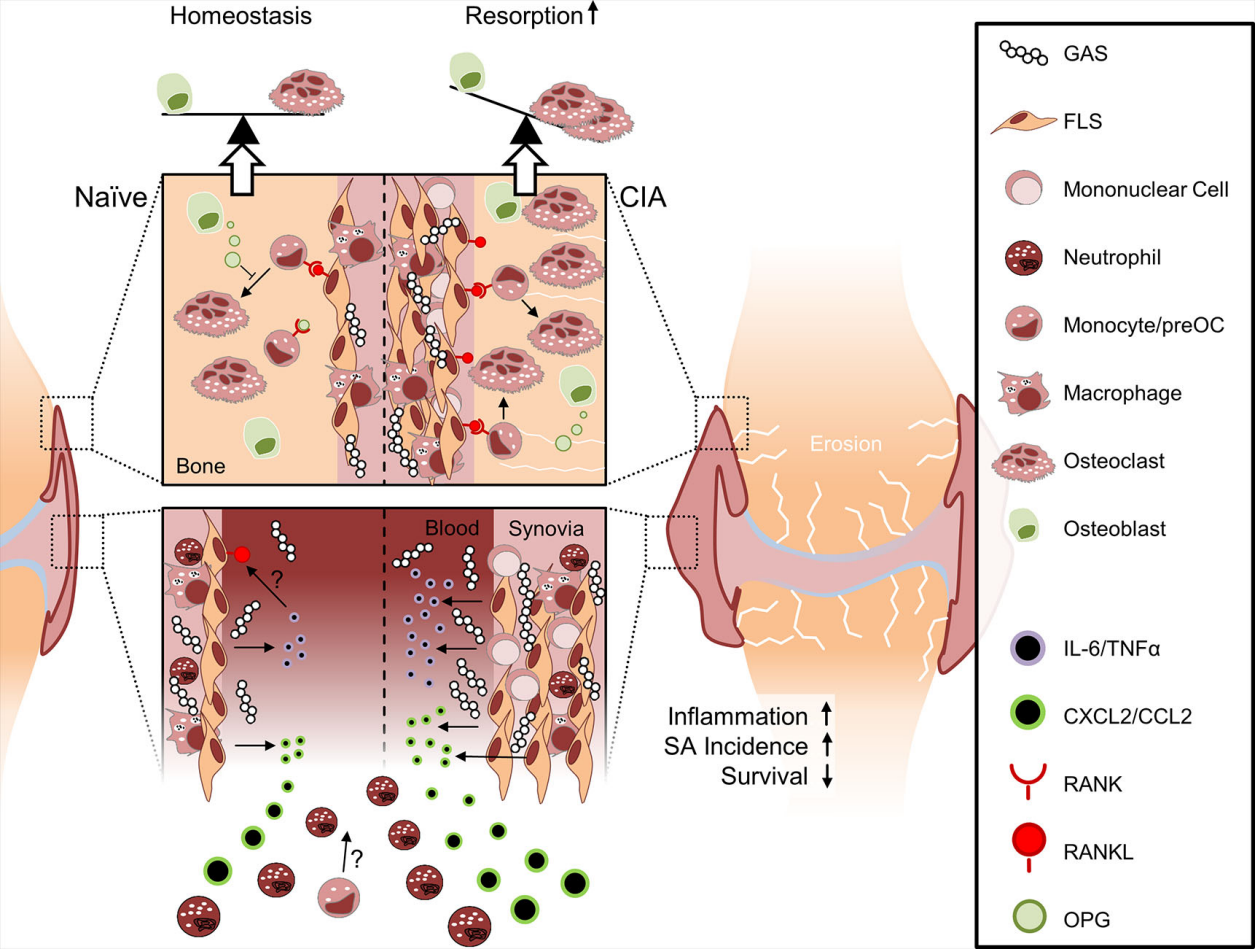
Collagen-induced arthritis facilitates sepsis and septic arthritis after Group A Streptococcus (GAS) infection: a model proposition. The synovial membrane of the naïve joint consists of a thin layer of fibroblast-like synoviocytes (FLS) and few macrophages. In collagen induced arthritis (CIA), however, FLS are highly proliferative and the synovial membrane is infiltrated by mononuclear cells. Compared to the naïve joint, the chronically inflamed synovia or pannus may facilitate the rapid discharge of increased amounts of cytokines and chemokines upon GAS encounter. Consequently, newly infiltrating neutrophils and possibly also monocytes further promote inflammation. Although it is not yet clear how CIA determines impaired bacterial clearance, the pre-existing disease entails a cytokines storm which is paralleled by an increased incidence of septic arthritis and a decreased survival probability. Certain sepsis-related cytokines, especially IL-6, are able to induce the presentation of RANKL on FLS at the interface between synovia and bone. RANKL consequently induces the differentiation of hematopoietic cells into osteoclasts. Bone forming osteoblasts can detain bone resorption by releasing OPG, a soluble RANKL surrogate, which impedes osteoclastogenesis. However, this homeostasis is vanquished by excessive RANKL expression that ultimately causes bone erosion.

In summary, SA in naïve mice led to acute inflammation that was insufficient to cause bone erosions. Mice with pre-existing CIA on the other hand were particularly susceptible to GAS infection and suffered from increased mortality and SA incidence. Aggravated SA was paralleled by excessive inflammation and significant bone destruction (**Figure 7**). Our results thus offer new insights into the interdependencies of different disease entities and point at inhibition of FLS activity in RA patients suffering from sepsis as a future potential avenue for intervention.

## Data Availability Statement

The raw data supporting the conclusions of this article will be made available by the authors upon reasonable request.

## Ethics Statement

The animal study was reviewed and approved by State Department for Agriculture, Food Safety and Fishery in Mecklenburg-Western Pomerania with the file reference number 7221.3-1.1-063/17.

## Author Contributions

The following are members of the KoInfekt Study Group: Michael Lalk (Institute of Biochemistry, University of Greifswald), Sophia Müller, and Erik Weipert (both Core Facility for Cell Sorting and Cell Analysis, University Medical Center Rostock). BM-H, MM, WB, and JV contributed to conception and design of the study. DS, MK, and JV performed the statistical analysis. JV wrote the first draft of the manuscript. BM-H and DS wrote sections of the manuscript. All authors contributed to the article and approved the submitted version.

## Funding

This research was funded by Federal Excellence Initiative of Mecklenburg-Western Pomerania and European Social Fund (ESF) Grant KoInfekt (ESF/14-BM-A55-0011/16 and ESF/14-BM-A55-0005/16).

## Conflict of Interest

The authors declare that the research was conducted in the absence of any commercial or financial relationships that could be construed as a potential conflict of interest.
