# Supplementary material for: Inflammatory Joint Disease Is a Risk Factor for Streptococcal Sepsis and Septic Arthritis in Mice
Source: Front Immunol. 2020 Oct 7;11:579475. doi: 10.3389/fimmu.2020.579475 (PMC7576673; doi:10.3389/fimmu.2020.579475)
Supplement: Supplementary file 1 [file DataSheet_1.docx]

Supplementary Material

##
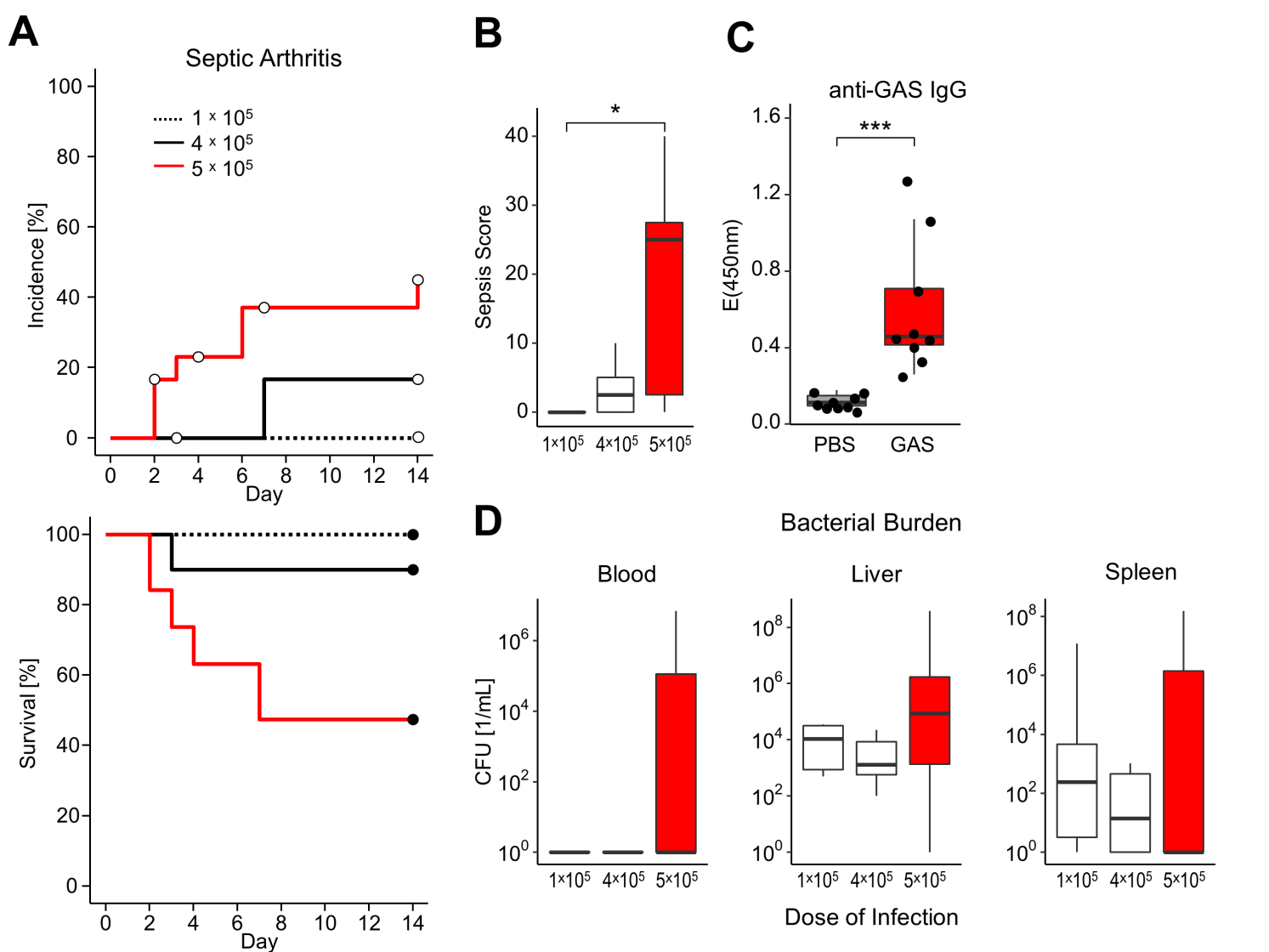


**Figure S1. Establishment of mouse model for sepsis and septic arthritis. (A)** C57BL/6 mice were infected with different doses of Group A Streptococcus (GAS) and were monitored for 14 days. Kaplan-Meier estimators of septic arthritis incidence and survival rate show that 5 ˣ 10^5^ colony-forming units of GAS are sufficient to induce septic arthritis in a broad range of infected animals. Mice were sacrificed at the end of the observation period or prematurely due to septic shock events. **(B)** Boxplots depicting sepsis scores at endpoint. **(C)** Murine anti-GAS IgG titers from mice infected with 5 × 10^5^ CFU GAS surviving until the end of the observation period. **(D)** Blood samples, liver and spleen homogenates were plated on blood agar to determine the bacterial burdens for each infection dose tested.


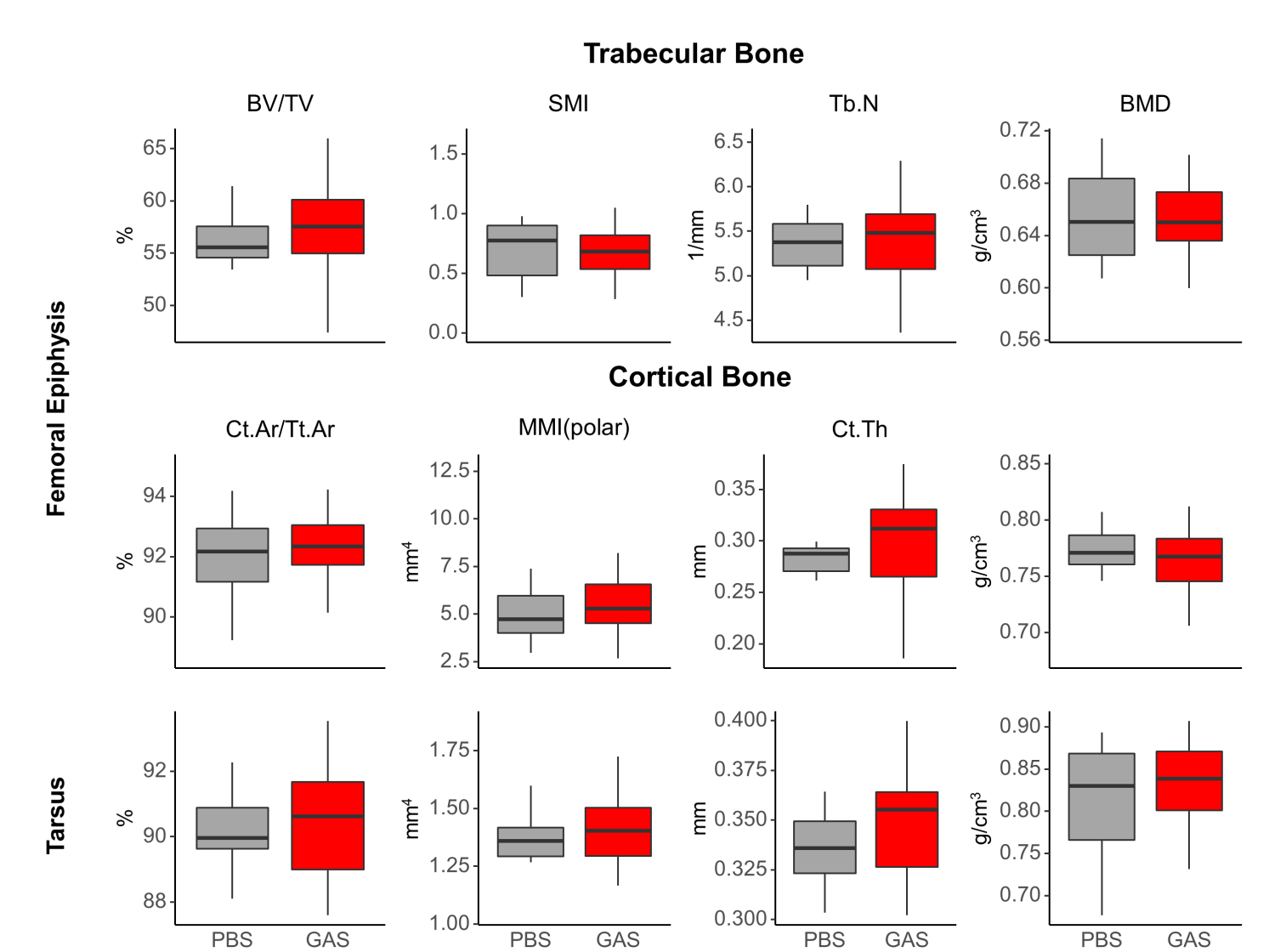


**Figure S2. Bone morphology after GAS infection. (A)** Femora and paws were analyzed by micro-computed tomography for changes in femoral and tarsal bone morphology parameters, respectively. Samples were taken at day 14 from PBS controls (n = 11). Samples from infected (GAS) mice were collected at days 3 (n = 6), 4 (n = 6) or 14 (n = 19). Bone volume fraction (BV/TV), structure model index (SMI), trabecular number (Tb.N) and bone mineral density (BMD) are shown for trabecular bone. Cortical area fraction (Ct.Ar/T.Ar), polar moment of intertia (MMI), cortical thickness (Ct.Th) as well as BMD is depicted for cortical bone. No significant changes were found but bone mass tends to increase as a result of the infection.


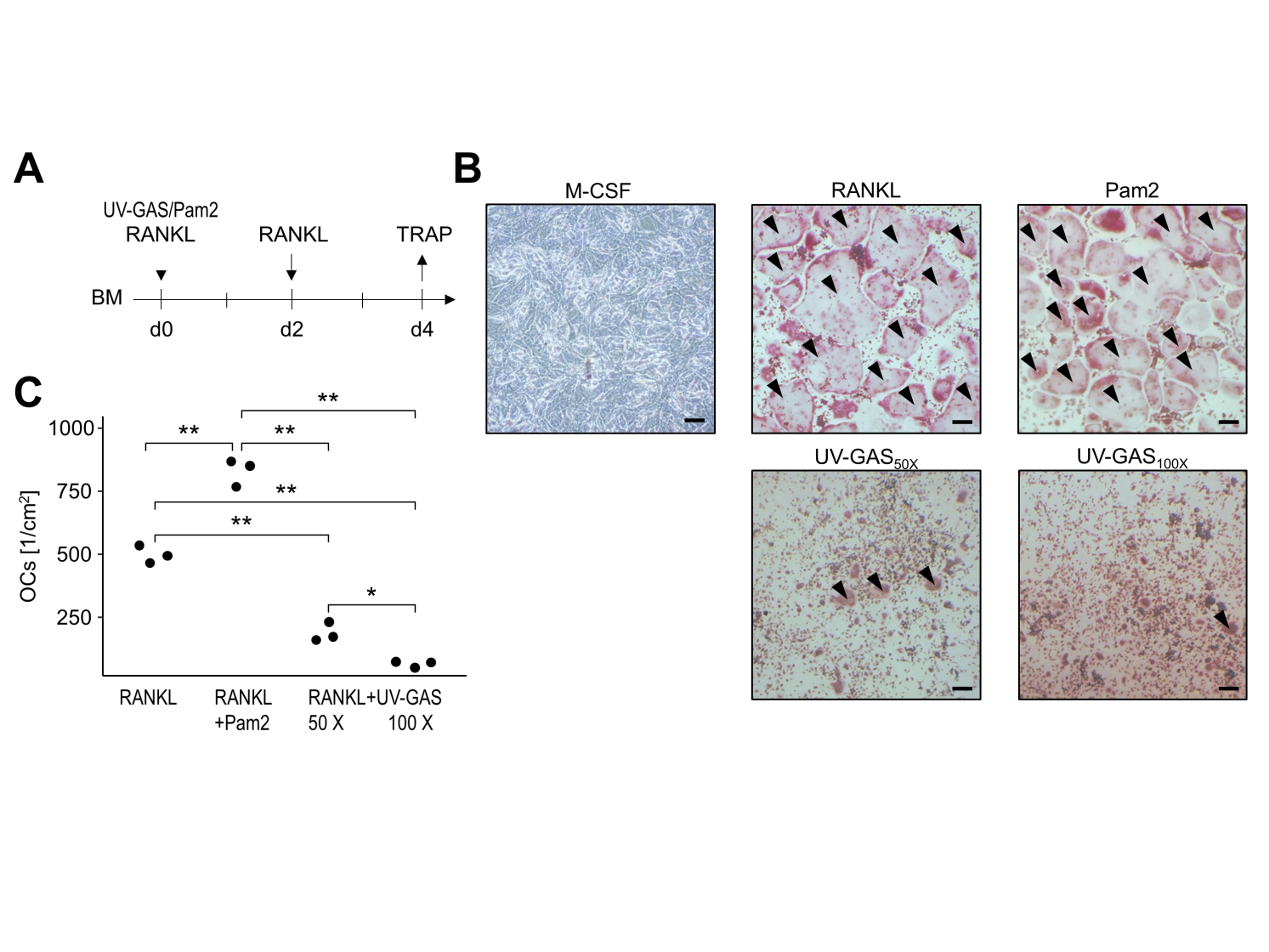


**Figure S3. Whole-bacteria GAS antigens inhibits RANKL-induced osteoclastogenesis. (A)** Experimental scheme showing the stimulation regimen. 2 × 10^5^ Bone marrow cells (BM) per cm^2^ from adult mouse long bones were extracted and cultivated in the presence of 30 ng/mL M-CSF, 50 ng/mL RANKL and UV-inactivated bacteria (UV-GAS) or 100 ng/mL of the TLR2 agonist Pam2CSK4 (Pam2) for the induction of osteoclastogenisis. UV-GAS were applied at a 50- and 100-fold amount relative to BM, respectively **(B)** Differentiation of bone marrow-derived macrophages to multinucleated osteoclasts (OCs) was visualized by staining of tartrate-resistant acidic phosphatase (TRAcP) and hematoxylin. TRAcP-positive cells with a nuclear count of ≥ 5 were considered osteoclasts (black arrowheads). Scale bars are 100 µm. **(C)** Dot plot showing the statistical analysis of osteoclasts counts normalized to area in the different culture regimens.

**
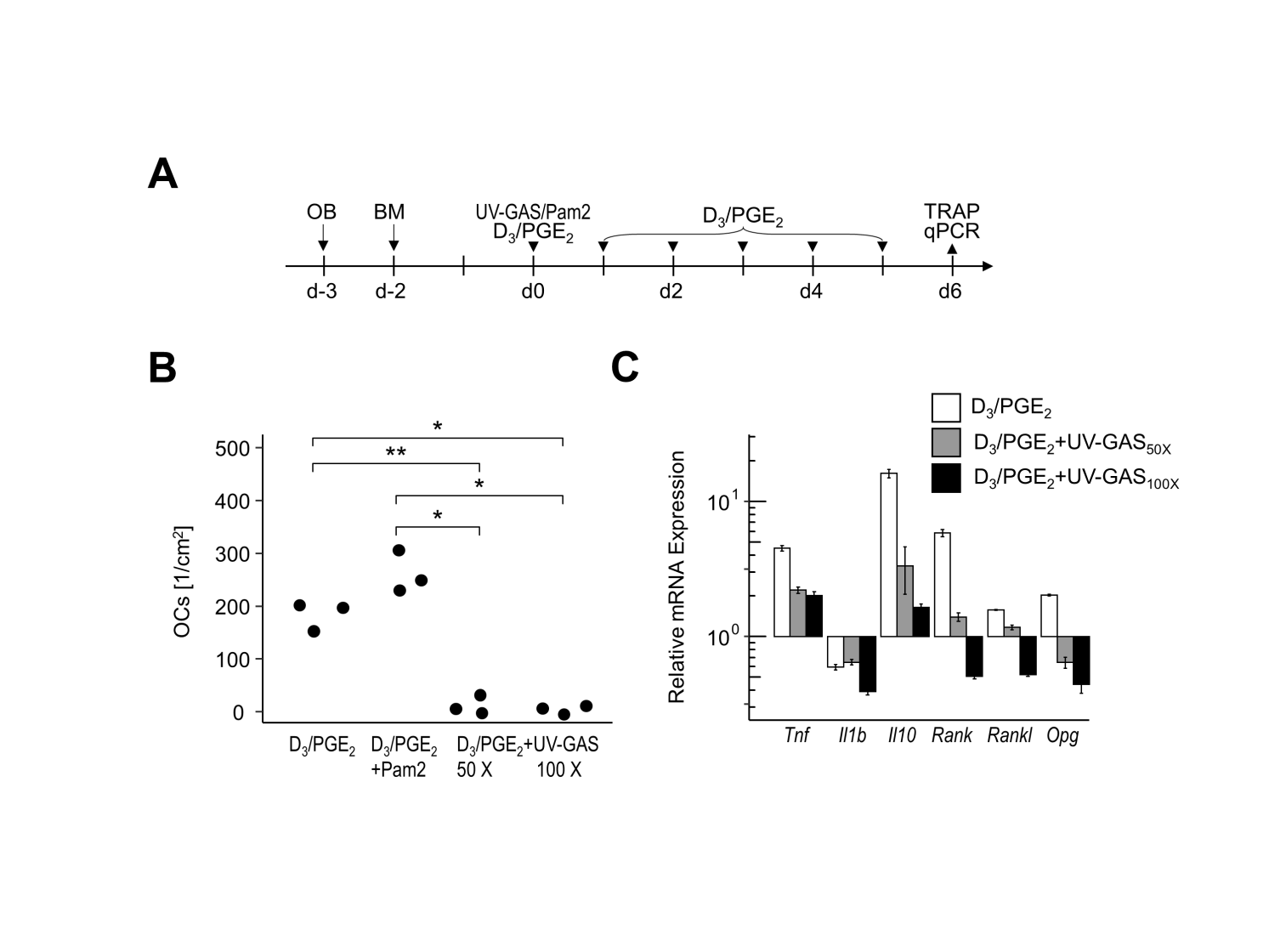
Figure S4. UV-inactivated GAS constrains osteoclast differentiation by distortion of the RANKL-RANK axis. (A)** Scheme of the co-culture experiment. Osteoblastogenic cells (OB) were extracted from neonatal mouse calvaria and were then co-cultured with bone marrow cells (BM) extracted from long bones at a 1:20 ratio. RANKL production by OB was triggered by the addition of 10 nm 1α,25-Dihydroxyviatmin (D_3_) and 1 µM prostaglandin (PG)E_2_. The TLR2 agonist Pam2CSK4 (Pam2, 100 ng/mL) and UV-inactivated GAS (UV-GAS) at a 50- or 100-fold amount relative to the eukaryotic cell count were added, respectively. **(B)** Multinucleated osteoclasts (OCs) were identified by TRAcP and hematoxylin staining of the co-culture 6 days after initial stimulation. **(C)** Relative mRNA expression from the culture bulk (n = 3) of different stimulation regimens was quantified for selected genes. Ct values were normalized to co-cultures whithout stimulation and the housekeeping gene *Gapdh* by the 2^-(ΔΔCt)^-Method. Means are depicted in a bar plot.


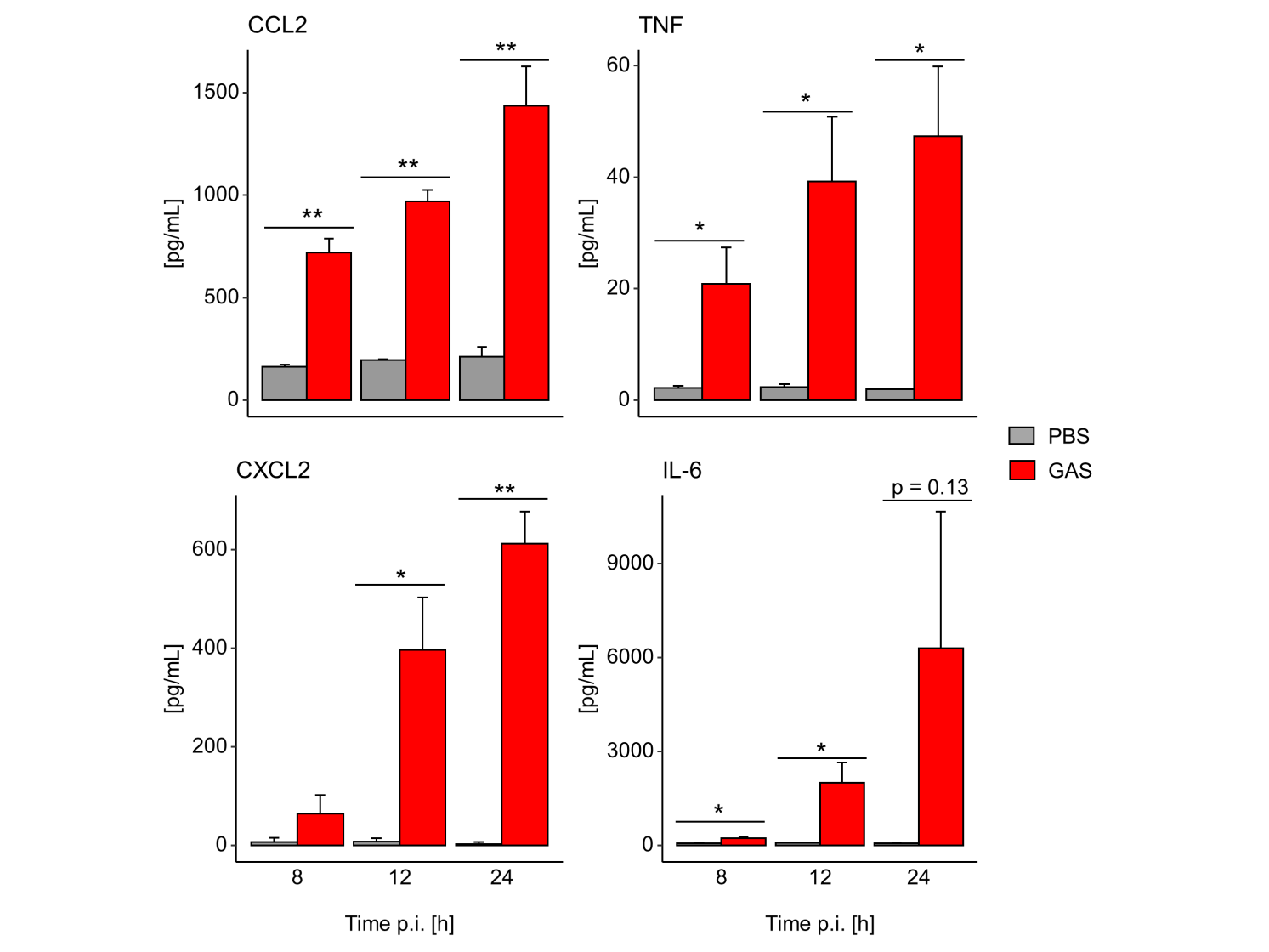


**Figure S5. GAS infection triggers FLS to upregulate chemokines and cytokines in a time-dependent manner.** Analyses of medium supernatants from FLS infected with GAS at a moi of 10 are shown as means + SD. Quantitative measurements were performed by ELISA and multiplexed bead-based flow cytometry assays, respectively, at 8, 12 or 24 h post infection. Gray bars show data from control cultures (n = 3) and red bars from infected cells (n = 3).


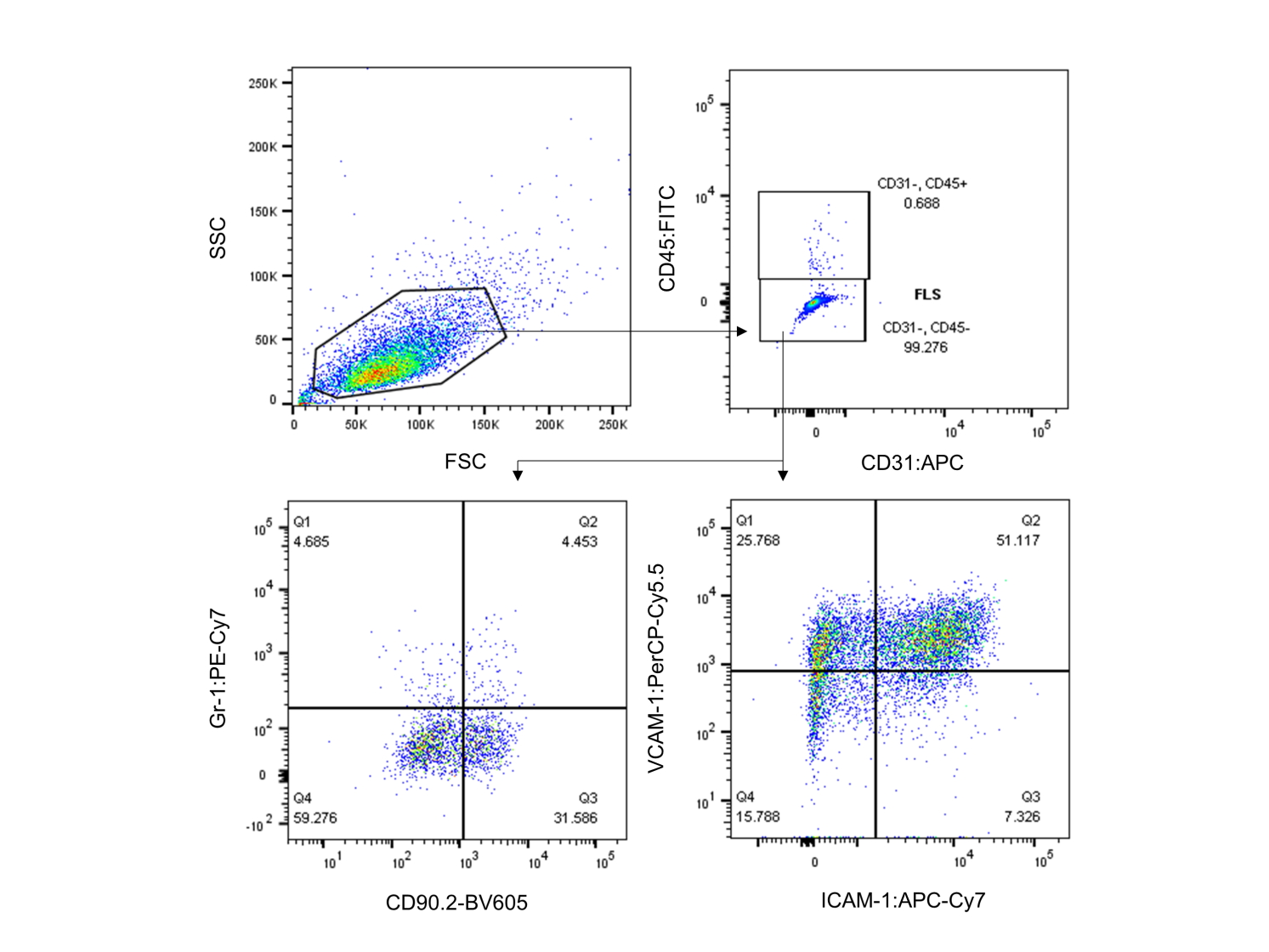


**Figure S6. Gating strategy for the characterization of fibroblast-like synoviocytes (FLS).** FLS were identified as CD31‾CD45‾ and can be furtherly divided into subpopulations expressing ICAM-1, VCAM-1, CD90.2 and/or Gr-1.


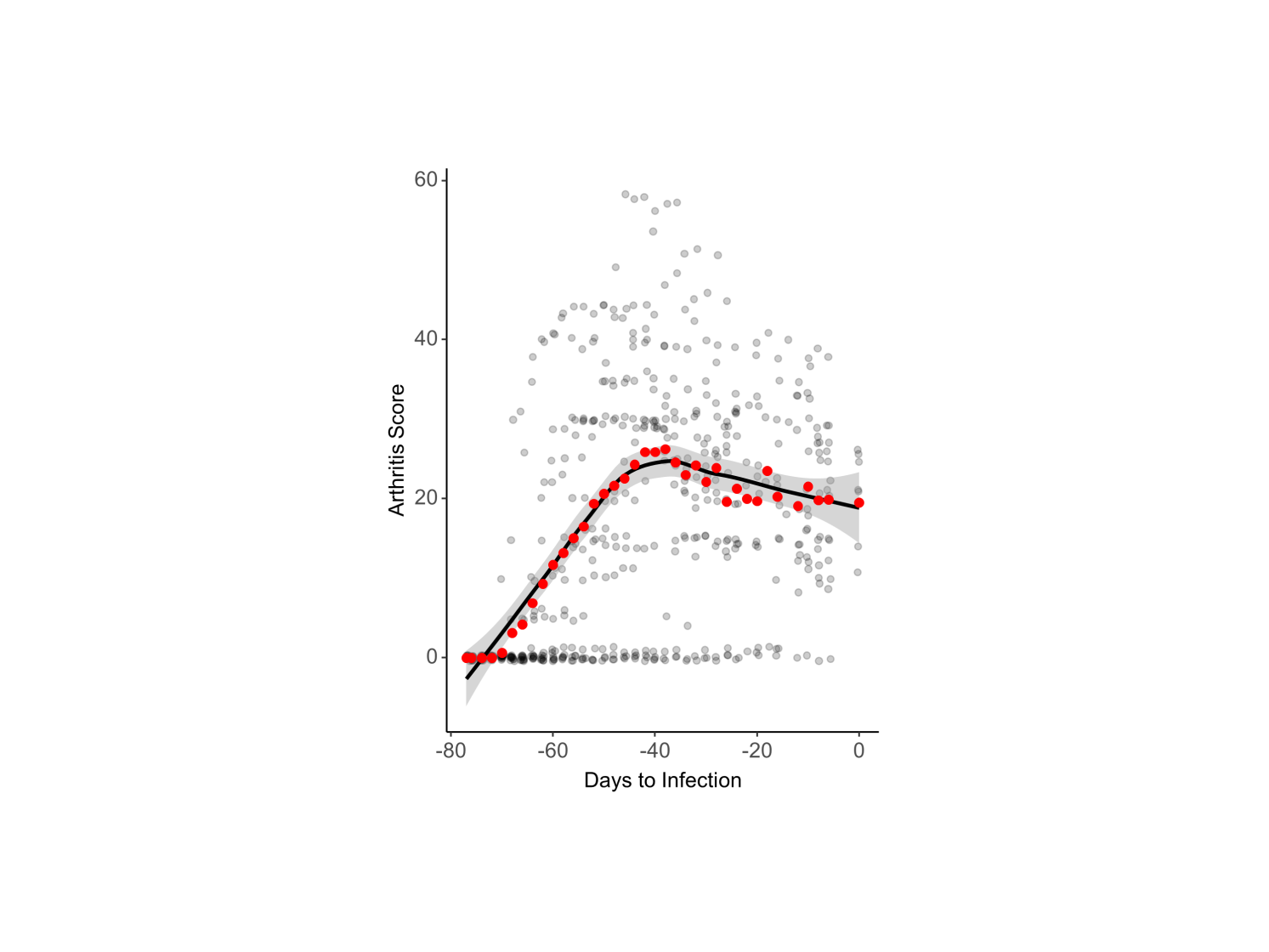


Figure S7. CIA enters remission before infection experiments. A total of 16 DBA/1 × B10.Q were immunized and boosted with bovine type II collagen 77 days before i.v. application of either PBS or 2 × 10^6^ CFU GAS. Individual arthritis scores (gray dots) and means of arthritis scores (red dots) are depicted in the scatterplot. Locally weighted scatterplot smoothing (black line) with a 0.95 confidence interval (gray area) was used to illustrate the decline of arthritis scores.


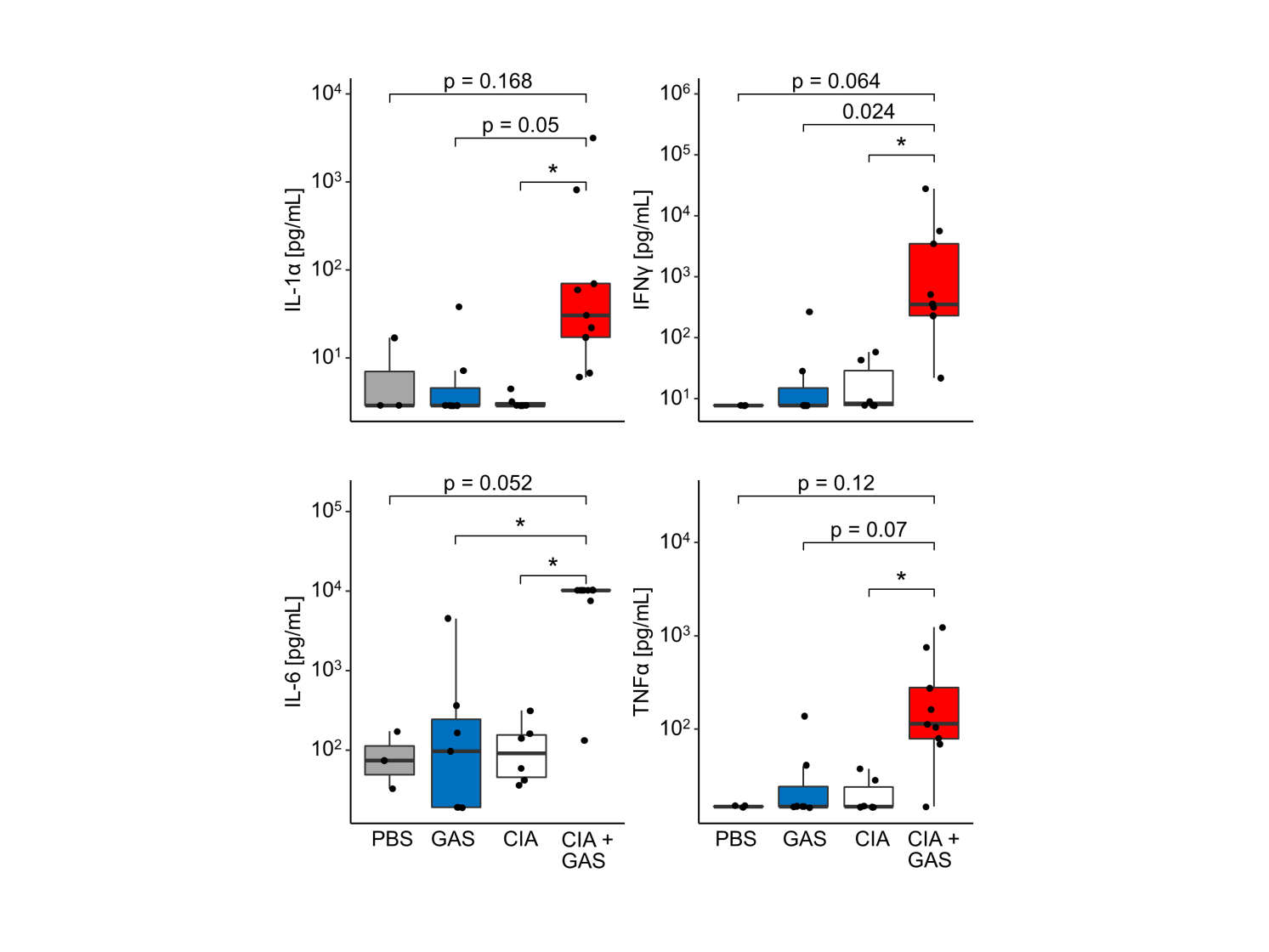


**Figure S8. Infection with GAS of mice with CIA leads to the hyper-responsive production of pro-inflammatory cytokines.** Cytokines from plasma samples were quantified by a multiplex flow cytometry approach at respective endpoints.


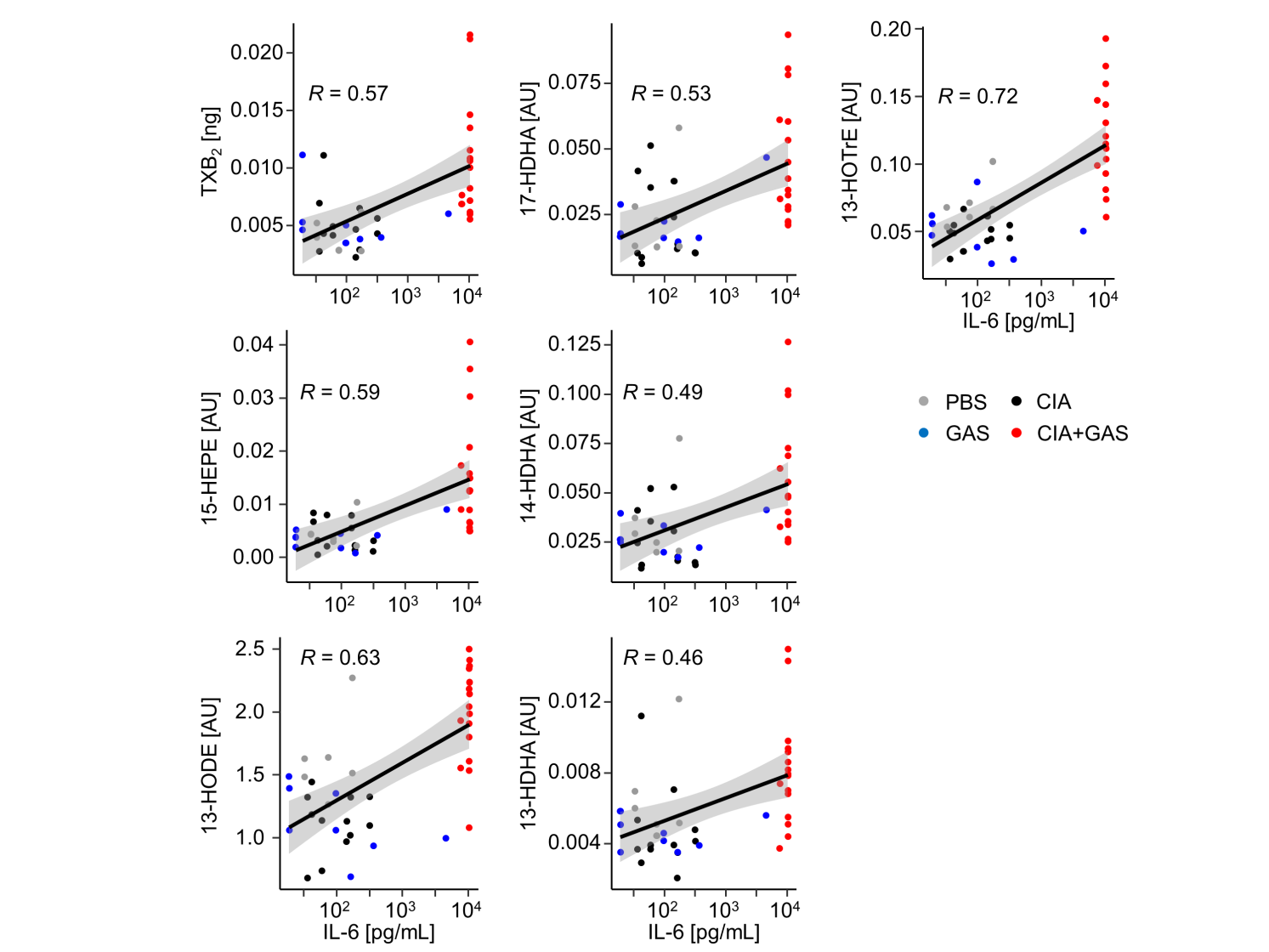


**Figure S9. Paw eicosanoids positively correlate with plasma Interleukin-6 concentrations in F1 mice.** Paw eicosanoids were determined by lipidomics using HPLC-MS/MS and were correlated plasma IL-6 concetrations. Gray areas depict the 0.95 confidence interval. *R*: Pearson product-moment correlation coefficient.
